# Analysis of fatty acid-derived lipids in critically ill patients after cardiac surgery yields novel pathophysiologically relevant mediators with possible relevance for systemic inflammatory reactions

**DOI:** 10.3389/fimmu.2024.1148806

**Published:** 2025-01-07

**Authors:** Holger Neb, Verena Roth, Jessica Roos, Tom Bauer, Anja Urbschat, Ulrike Heinicke, Carlo Angioni, Dieter Steinhilber, Matthias Piesche, Nerea Ferreirós, Robert Gurke, Gerd Geisslinger, Edith Utech, Kai Zacharowski, Patrick Meybohm, Patrick Paulus, Elke Schmitt, Thorsten Jürgen Maier

**Affiliations:** ^1^ Department of Anesthesiology, Intensive Care Medicine and Pain Therapy, Frankfurt University Hospital, Goethe University Frankfurt, Frankfurt, Germany; ^2^ Department of Immunology, Paul-Ehrlich-Institute (Federal Institute for Vaccines and Biomedicines), Langen, Germany; ^3^ Department of Biomedicine, Aarhus University, Aarhus, Denmark; ^4^ Institute of Clinical Pharmacology, Frankfurt University Hospital, Frankfurt, Germany; ^5^ Institute of Pharmaceutical Chemistry, Goethe University Frankfurt, Frankfurt, Germany; ^6^ Fraunhofer Institute for Translational Medicine and Pharmacology (ITMP) and Fraunhofer Cluster of Excellence for Immune Mediated Diseases (CIMD), Frankfurt, Germany; ^7^ Biomedical Research Laboratories, Medicine Faculty, Universidad Católica del Maule, Talca, Chile; ^8^ Oncology Center, Medicine Faculty, Universidad Católica del Maule, Talca, Chile; ^9^ Department of Anesthesiology, Intensive Care, Emergency and Pain Medicine University Hospital Wuerzburg, Wuerzburg, Germany; ^10^ Laboratory for Experimental Anesthesiology, Johannes Kepler University (JKU), Linz, Austria, Department of Anesthesiology and Operative Intensive Care Medicine, Kepler University Hospital Linz, Linz, Austria; ^11^ Institute of Biostatistics and Mathematical Modelling, Department of Medicine, Goethe University Frankfurt, Frankfurt, Germany

**Keywords:** eicosanoids, prostaglandins, sphingolipids, ceramides, endocannabinoids, systemic inflammation, biomarker

## Abstract

**Introduction:**

Critically ill patients suffer from a wide variety of clinical events, most of them leading to pro-inflammatory states such as sepsis or simply as consequence of major surgery. Many of these patients develop forms of acute kidney injury, heart or acute liver failure during intensive care. Lipid signaling is critically involved in triggering systemic inflammation processes, pain and vascular tone. We therefore hypothesized that fatty-acid-derived lipid mediators might be regulated during inflammatory stages and other clinical events in critically ill patients and might serve as potential biomarker candidates.

**Methods and study design:**

Using liquid chromatography-tandem mass spectrometry (LC-MS/MS), we determined the levels of 53 lipid mediators in plasma from nine patients. These patients were hospitalized at Frankfurt University Hospital’s intensive care unit (ICU) after cardiac surgery. Inflammatory stages were illustrated over time using clinically established biomarkers such as interleukin-6 (IL-6) and leukocyte count. Normal range values of the lipids were obtained from healthy volunteers.

**Results:**

Plasma levels clearly outside the normal range were observed for 22 of 53 lipid mediators, of which 13 were increased (including ceramides Cer (d18:0/18:0), Cer (d18:1/16:0), Cer (d18:1/18:1), glucosyl-ceramide GluCer (d18:1/24:1), lactosylceramide LacCer (d18:1/18:0), and LacCer (d18:1/24:1), 6-keto-prostaglandin F1alpha (6-keto-PGF1alpha), 11,12- and 14,15-DHET and 1- and 2-arachidonoyl glycerol (1-AG and 2-AG), Sphingosine SPH (d18:1) and 20-HETE. Furthermore, nine lipids were decreased (Cer (d18:1/24:0), LacCer (d18:1/16:0), LacCer (d18:1/24:0), sphingosine-1-phosphate S1P (d18:1), S1P (d18:0), the lysophosphatidic acids LPA (16:0), LPA (18:0), LPA (18:1) and 9-HODE. Among increased lipids, the remarkable changes in 1-AG, 2-AG, and to a lower extent of 6-keto-PGF_1-alpha_ plasma levels showed a certain agreement with inflammatory phases. Furthermore, 6-keto-PGF1_alpha_ had its peak shortly before initiation of continuous veno-venous hemodialysis (at least in 5 of the observed patients), 2-AG was elevated in all our nine patients during (right) heart failure in the context of either re-opening patient’s chest, implementation of veno-arterial ECMO or at least while significantly increasing the amount of catecholamines.

**Discussion:**

In this pilot trial we identified several evaluated lipids in critically ill patients representing either potentially (patho-) physiologically relevant mediators of the pro-inflammatory processes and during heart failure or possible markers preceding veno-venous hemodialysis.

## 1 Background

Patients in critical conditions may experience a wide range of pro-inflammatory clinical events, the majority of which result from major surgery or as result of infection-associated complications like sepsis. Following a lengthy and intricate surgery, individuals, particularly those undergoing heart surgery, are doomed to experience acute systemic inflammation. A number of these patients under inflammatory conditions develop acute liver failure, acute cardiac damage, or acute renal injury.

**Figure 1:**
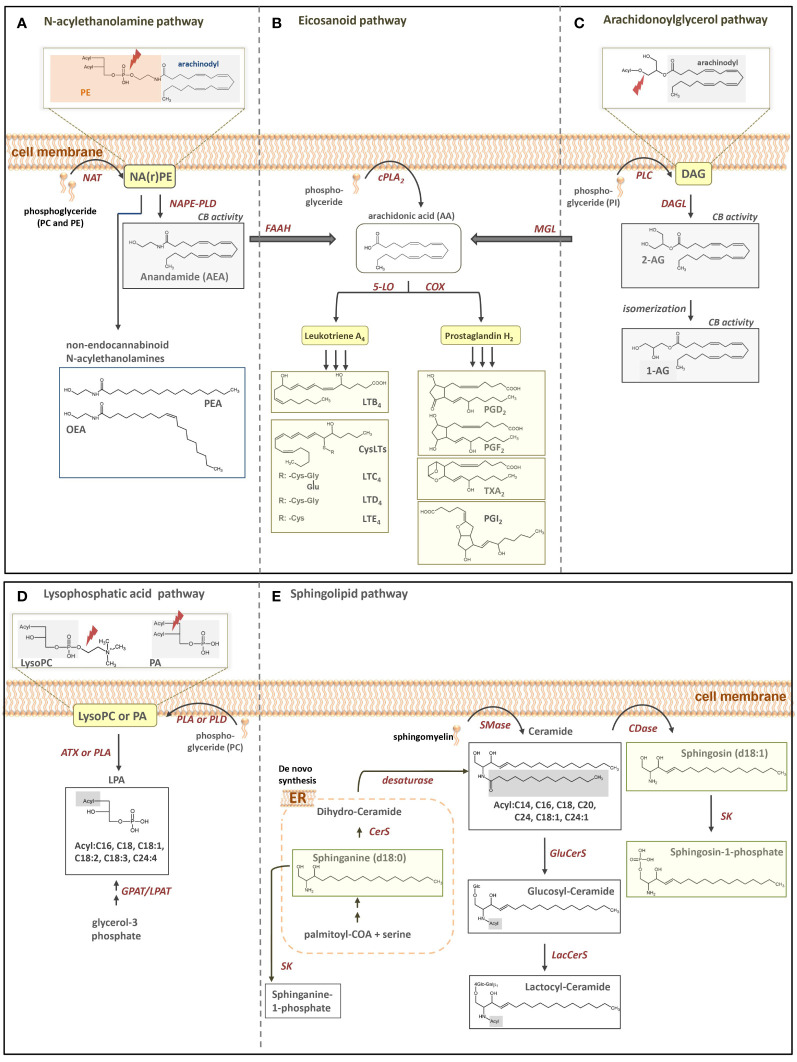
Simplified illustration of fatty acid-derived lipid biosynthesis pathways and products investigated in our study. **(A)** N-acylethanolamine, **(B)** eicosanoid, **(C)** arachidonoylglycerol, **(D)** LPA and **(E)** sphingolipid pathway. PE, phoaphatidylethanolamine; NAPE, N-acyl-phosphatidylethanolamine; NArPE, N-arachidonoyl-phosphatidylethanolamine; NAPE-PLD, NAPE-selective phospholipase D; NAT, N-acyltransferase; PEA, palmitoylethanolamine; OEA, oleoylethanolamine; FAAH, fatty acid amide hydrolase; cPLA2, cytosolic phospholipase A2; 5-LO, 5-lipoxygenase; COX, cyclooxygenase; PG, prostaglandin; LT, leukotriene; PI, phosphatidylinostiol; DAG, diacylglycerol; DAGL, diacylglycerol lipase; AG, arachidoylglacerol MGL, monoacylglycerol lipase; LysoPC, lysophosphatidylcholine; PA phosphatic acid; PC, phosphatidylcholine; LPA, Lysophosphatic acid; GPAT, glycerol-3-phosphate acyltransferase; LPAT, lysophosphatidic acid acyltransferases; PLA, phospholipase A, PLD, phospholipase D; ATX, autotaxin; ER, endoplasmatic reticulum; CerS, Ceramide synthase; Smase, sphingomyelinase; Cdase, ceramidase; SK, sphingosine kinase; GluCerS, glucosylceramide synthase; LacCerS, lactocylceramide synthase.

Bioactive lipid mediators deriving from the arachidonic acid cascade and members of the sphingolipid family have shown to play a crucial role in inflammatory processes (1–4). Plasma levels of certain lipid mediators might therefore work as potential new biomarker candidates to early and reliably indicate changes in clinical stages as well as pathophysiological relevant mediators, triggering or counteracting specific inflammatory stages. As an example, S1P (d18:1) plays an important role in maintaining endothelial integrity, and Winkler et al. already concluded that low S1P (d18:1) levels might crucially contribute to capillary leakage (1). Taking the example of sepsis as specific cause for a pro-inflammatory state of patients’ immune system, different lipid mediators have been discussed as crucial or at least taking part in the course of the disease. In these cases, synthetization of inflammatory arachidonic acid pathway metabolites such as prostaglandin E2 (PGE_2_) is reduced, pointing to a worse clinical outcome (5). Moreover, the generation of 5-lipoxygenase pathway product leukotriene C_4_ (LTC_4_) is significantly reduced during sepsis and elevated LTC_4_ production was associated with higher patient survival. For reader’s overview, **Figure 1** illustrates the simplified biosynthesis pathways of some relevant lipid mediators of the eicosanoid and sphingolipid families, which have been investigated in the present study.

To this date, only a few studies have focused on fatty acid-derived lipids as potential biomarkers indicating inflammatory stages (6–8). Possibly due to methodological and analytical limitations, previous studies analyzed serum levels of lipids at definite time points. To the best of our knowledge, no study has analyzed a broad range of lipid profiles over extended time periods with lipid plasma level analysis following the patients stay at the ICU for several days. Therefore, the present study was aimed at simultaneously analyzing 53 lipid-signaling mediators from the plasma of nine patients hospitalized at Frankfurt University Hospital ICU for a period up to 28 days. We conclude that some lipid mediators may serve for early detection of new ongoing inflammation processes or might give insights to distinguish between different entities of pro-inflammatory states. To compare inflammatory reactions in patients with frequently used *ex vivo* models of inflammation, lipid mediator levels were compared to plasma lipid levels of healthy volunteers, stimulated *ex vivo* with the bacterial toxin lipopolysaccharide (LPS). Here, we present several lipid mediator-signaling pathways significantly regulated during periods of inflammation caused by different clinical events after prolonged cardiac surgery.

## 2 Materials and methods

### 2.1 Study design and sample collection

Cardiac surgery patients, who underwent either at least re-operation, double valve repair or replacement and large blood vessel surgery with complete cardio-pulmonary bypass (cbp) were included. Furthermore, patients were included where surgery was planned with extra-long cbp time. We aimed for patients with a suspected vigorous inflammatory response and a narrow age window. Nine ICU patients (see **Table 1**) were finally included based on the following criteria: age > 18 years and < 80 years, post-operative stay at ICU for at least 8 days and more than 8 blood collections for lipid mediator analyses. Patients’ characteristics including further information on treatments can be found in **Table 1**. Samples including those for *ex vivo* experiments were collected in the period 2013–2020 at the Department of Anesthesiology, Intensive Care Medicine and Pain Therapy, Frankfurt University Hospital, Goethe University Frankfurt, Germany. In this pilot trial, the number of patients included was restricted by limited analytical capacity of LC/MS-MS, given the numerous lipid mediators to be analyzed daily over prolonged periods. Study-related blood drawing for determining IL-6 and leukocyte count was performed twice daily. Blood drawing for lipid mediator analysis was performed once daily for an average of 17.2 days (min. 8 days, max. 28 days.) Whole blood samples were collected daily from the included patients, starting on the first day after surgery. As the physiological and pathophysiological functions of most of the investigated 53 lipids have not yet been well characterized, their normal ranges in human plasma are largely unknown. The 95% and 2-fold upper and lower 95% normal range values of the lipids were therefore determined via LC-MS/MS analysis of the lipid mediator concentrations in plasma samples from ten healthy volunteers, who neither suffered from inflammatory diseases that could theoretically impact lipid plasma levels nor were under any drug therapy. Notably, due to unexpected technical limitations, not all normal lipid range values (38 out of 53 lipids) could be determined with the first blood sample collection. Therefore, a second blood sample collection and LC-MS/MS analysis, involving a further ten healthy volunteers, was conducted for the normal range determination of the 15 remaining plasma lipid levels, including eicosatetraenoic acids (HETEs), eicosatrienoic acids (DHET), phosphoglycerides (LPAs), epoxides of linoleic acid (EpOME), diols of linoleic acid (DiHOMEs) and hydroxyoctadecadienoic acids (HODEs). Prior to LC-MS/MS analysis, all person-related data in this study, except age and gender, were pseudo-anonymized. Lastly, the patients’ plasma lipid mediator levels were compared with those obtained from the stimulation of venous whole blood samples from the first healthy volunteer group using the bacterial component lipopolysaccharide (LPS) (see section 2.4). This experiment was aimed at addressing the question to what extent *ex vivo* models of inflammation reflect the *in vivo* situation of patients accurately and to what extent lipid mediator reactivity under pro-inflammatory conditions is similar among different patients and models of inflammation.

**Table 1:** Overview of patients included in the study.

patient number	gender	Pre-existing conditions ^a^	age (years) at ICU admit-tance	duration of ventilation (hours)	Death on ICU	catelcholamine used ^b^	SOFA ^a^ Score at admission	Renal Replacement Therapy	Lactat at admission [mg/dl]
1	female	AVD, MVD, AHT, T2D, HT, CHD with stent, c.a. B Ca, CKD, HLP	66	335	no	norepinephrine, epinephrine, milrinone, vasopressin	12	yes	32
2	male	AVD, cAA, T2D, AF, c.a. ACVB, HT, COPD	60	216	no	epinephrine, vasopressin, norepinephrine, milrinone	13	no	40
6	male	AVD, HT, AB, CHD, MH, c.a. BL Ca	68	190	no	norepinephrine, milrinone, vasopressin	12	no	42
8	male	AVD, AA, AHT, COPD, AF, CHD	64	165	no	norepinephrine, epinephrine, milrinone, vasopressin	11	no	11
9	female	CKD, COPD, OSAS, MI with c.a. CPR, ischaemic CDM, VA and OBS	58	485	yes	norepinephrine, milrinone	8	no	7
10	male	CHD, T2D, COPD	69	168	no	norepinephrine, epinephrine, milrinone, vasopressin	9	no	34
11	male	MI with acute HF, HT, CKD	45	480	yes	norepinephrine, epinephrine, milrinone, vasopressin	11	yes	30
14	male	CHD, c.a. MI, CDM, T2D, HLP, PAD, AHT, CKD	65	489	yes	norepinephrine, epinephrine, milrinone, vasopressin	14	no	39
15	male	CHD, c.a. MI, CDM, AHT, PAD, CKD, gout, adiposity	67	157	yes	norepinephrine, epinephrine, vasopressin	15	No	105

a Explanation of abbreviations: AA, aortic aneurysm; cAA, chronic anemia; AB, asthma bronchiale; ACVB, aortocoronary venous bypass; AF, atrial fibrillation; AHT; arterial hypertension; AVD, aortic valve disease; B Ca, breast cancer; BL Ca, bladder cancer; c.a., condition after; CDM, cardiomyopathy; CHD, coronary heart disease; CKD, chronic kidney disease; COPD, chronic obstructive pulmonary disease; CPR, cardiopulmonary resuscitation; HF, heart failure; HLP, hyperlipidemia; HT, hypothyroidism; MH, Morbus Hodgkin; MI, myocardial infarct; MVD, mitral valve disease; OBS, organic brain syndrome; OSAS, obstructive sleep apnea syndrome; PAD, atherosclerotic peripheral artery disease; SOFA, sequential Organ Failure Assessment; T2D, typ 2 diabetes; VA, ventricle aneurysm.

b During the acute disease phase.

### 2.2 Assessment of ICU patients’ clinical parameters

Clinical data were retrospectively collected from our clinical ICU database (MetaVision, iMDsoft, Israel), including routine diagnostic laboratory data (LAURIS, Nexus, Germany) and our imaging data system (PACS, Siemens Healthcare, Germany). A senior medical doctor screened every patient’s history mainly for signs of general inflammation, hemodynamic instability, progress in acute kidney injury and acute liver failure. Mainly, inflammation was measured using IL-6 as a significant diagnostic marker (9, 10). In addition, to enable differentiation of septic phases from inflammatory reactions due to severe post-operative organ damage mainly resulting from left or right heart failure, special attention was given to microbiological test results (see **Table 2**).

**Table 2:** Inflammatory stages and possible association with clinical events.

patient number	time since ICU admittance (hours)	Possibe event associated with inflammation	Microbial test results
1	20	surgery: aortic valve replacement	
	100	ECMO^b^ implantation	
	230	ECMO^b^ explantation	
	300	bacterial pneumonia	Staph Epi. (G pos.); Pseud. Aer. (G neg.); C. Diff. (G pos.); VRE (G pos.); Serratia Mar. (G neg.); Cand. Glab.; Bacteroides spec. (G neg.)
2	20	surgery: aortic valve replacement, ACVB^c^	
	60	MOF^d^, cause unknown	
	340	bacterial peritonitis	Staph. Epi. (G pos.); E. Coli (G neg.)
6	20	surgery: aortic and mitral valve replacement	
	40	bacterial pneumonia	Pseudo. Aer. (G neg.)
8	20	surgery: aortic valve replacement, ACVB^c^	Klebs. Pneum. (G neg.); VRE (G pos.)
9	14	surgery: ventricle reconstruction, bacteremia focus unknown	Staph. Hämolyt. (G pos.); E. Faecium (G pos.); Ent. Cloacae (G neg.); Fusarium Oxysporum
	40	mechanical CPR^e^	
	250	pericardial effusion: re-surgery	
	330	candidemia, focus unknown	Candida Alb.
10	0	surgery: mitral valve replacement, ACVB^c^	
	50	cardiogenic shock	
	160	bacterial pneumonia	Citrobac. Freundii (G neg.); Pseud. Aer. (G neg.)
11	30	surgery: LVAD^f^ implantation, followed by pericardial effusion with re-surgery	
	240	primary peritonitis	
	310	primary peritonitis, bacterial pneumonia	Acinet. Baumanii (G neg.)
	530	colitis, bacterial pneumonia	
	660	candida pneumonia	Cand. Glab.
14	20	surgery: ACVB^c^	E. Faecalis (G pos.); Bacteroides Spec. (G neg.); Candida Glabr.; Pseud. Aer. (G neg.); E. Faecium (G pos.); Kleb. Pneum. (G neg.); Staph. Epi. (G pos.)
	50	mechanical CPR^e^	
	270	mechanical CPR^e^	
15	30	Surgery: ACVB^c^ plus bacterial pneumonia	VRE (G pos.); Staph Epi. (G pos.); E.coli (G neg.); E.faecium (G pos.); Strept.mitis (G pos.)
	210	bacterial pneumonia	
	290	candida peumonia, primary peritonitis	Cand. Glab.

a Some patients had infections with several germs at the same time. The location of sampling were central catheters, BALs, urinary sampling, urinary catheters, stool samples and blood cultures. However, the exact location of every germ found is not precise.

b Extracorporeal membrane oxygenation.

c Aortocoronary venous bypass.

d Multiple organ failure.

e Cardiopulmonary resuscitation.

f Left ventricular assist device.

We further screened especially for other disturbing factors which can potentially disrupt lipid levels such as different medical histories, co-medications with diverse pharmacotherapeutic treatment regimens and medical-technical interventions like the beginning of dialysis, artificial respiration and extracorporeal life support.

### 2.3 Sample recovery to determine ICU patients’ plasma lipid levels and healthy volunteers’ normal lipid ranges

Citrated venous whole blood was collected daily at the same time point from nine ICU patients hospitalized at Frankfurt University Hospital and immediately centrifuged at 2000 g at 4°C for 15 minutes. Supernatants (plasma) of patients and samples of healthy volunteers for determination of the normal range were stored at -80°C before LC-MS/MS analysis. We used a sensitive LC-MS/MS method to determine 53 lipid mediators (**Table 3**) originating from different lipid signaling pathways.

**Table 3:** Lipid mediators (n=53) analyzed in the present study using LC-MS/MS.

Sphingolipids (22)	
Ceramides	Cer (d18:0/16:0) Cer (d18:0/18:0) Cer (d18:0/24:0) Cer (d18:1/14:0) Cer (d18:1/16:0) Cer (d18:1/18:0) Cer (d18:1/18:1) Cer (d18:1/20:0) Cer (d18:1/24:0) Cer (d18:1/24:1) Cer (d18:0/24:1)
Glucosylceramides	GlcCer (d18:1/16:0) GlcCer (d18:1/18:0) GlcCer (d18:1/24:1)
Lactosylceramides	LacCer (d18:1/16:0) LacCer (d18:1/18:0) LacCer (d18:1/24:0) LacCer (d18:1/24:1)
Sphinganines	Sphinganine SPH (d18:0) Sphinganine-1-phosphate S1P (d18:0)
Sphingosines	Sphingosine SPH (d18:1) Sphingosine-1-phosphate S1P (d18:1)
Eicosanoids (19)	
Leukotrienes (LT)	Leukotrien B_4_ (LTB_4_)
Prostaglandins (PG)	PGE_2_ PGD_2_ PGF2alpha
Prostacyclins	6-Keto-PGF1alpha
Prostanoids	Thromboxane B_2_
Hydroxyeicosatetraenoic acids (HETE)	5-HETE 12-HETE 15-HETE 20-HETE
Dihydroxyeicosatrienoic acid (DHET)	5,6-DHET 8,9-DHET 11,2-DHET 14,15-DHET
Endocannabinoids	*N*-Arachidonoyl ethanolamide (AEA) Oleoylethanolamide (OEA) *N*-Palmitoylethanolamide (PEA) 1-Arachidonoylglycerol (1-AG) 2-Arachidonoylglycerol (2-AG)
Phosphoglycerides (6)	
Lysophosphatidic acid (LPA)	LPA (16:0) LPA (18:0) LPA (18:1) LPA (18:2) LPA (18:3) LPA (20:4)
Further fatty acid derivatives (6)	
Epoxides of linoleic acid (EpOME)	9,10-EpOME 12,13-EpOME
Diols of linoleic acid (DiHOME)	9,10-DiHOME 12,13-DiHOME
Hydroxyoctadecadienoic acids (HODE)	9-HODE 13-HODE

### 2.4 *Ex vivo* whole blood LPS assay

The venous whole blood samples of ten healthy male and female voluntary test subjects aged ≥18 served for plasma lipid profile assessment after ex vivo stimulation with the bacterial component lipopolysaccharide (LPS). For the *ex vivo* assay, 1 mL heparinized whole blood was incubated with LPS (100 ng/ml) for 8, 16, and 24 h at 37°C under gentle stirring (180 rpm). We have chosen 100 ng/ml LPS as it represents a commonly used low but already sufficient concentration to trigger the release of a broad spectrum of pro-inflammatory mediators, including both lipid mediators and cytokines (e.g (11)). Notably, a broad range of lipids were analyzed after LPS treatment with a number of them playing well-documented roles in inflammation. However, LPS as stimulus cannot trigger stimulation/repression of biosynthesis of all lipid mediators. Some lipids analyzed in this study may not be involved in inflammation with a mode of regulation of biosynthesis not fully understood. Samples without LPS (0 h) were used as a control for the ex vivo LPS stimulation assay. Native control plasma of these volunteers also served to determine the normal ranges of the first series of 38 lipids investigated in this study.

### 2.5 LC-MS/MS analysis of lipid mediator levels in patients’ and healthy volunteers’ plasma to determine normal ranges and in plasma obtained from LPS-stimulated whole blood

All lipids were analyzed via liquid chromatography-electrospray ionization-tandem mass spectrometry (LC-ESI-MS/MS) at the Institute of Clinical Pharmacology, Frankfurt University Hospital, Frankfurt am Main, Germany.

#### 2.5.1 Analysis of endocannabinoids

Analysis of arachidonoylethanolamide (AEA), palmitoylethanolamide (PEA), 1- and 2-arachidonoylglycerol (1- and 2-AG), and oleoylethanolamide (OEA) was carried out as described elsewhere (12). Briefly, 50 µL plasma was liquid–liquid extracted. The residues were reconstituted with 50 µL of acetonitrile in glass vials, and 10 µL were injected into the LC-MS/MS system. This consisted of a hybrid triple quadrupole-ion trap QTrap 5500 mass spectrometer (Sciex, Darmstadt, Germany) equipped with a Turbo-V-source operating in negative ESI mode, an Agilent 1200 binary HPLC pump, column oven (40°C), and degasser (Agilent, Waldbronn, Germany), and an HTC Pal autosampler (Chromtech, Idstein, Germany). A cooling stack was used to store the samples at 4°C in the autosampler. HPLC analysis was carried out under gradient conditions using a Luna C18 column (150 mm L×2 mm ID, 5 µm particle size, Phenomenex, Aschaffenburg, Germany) and water and acetonitrile, both containing 0.01% ammonia as mobile phases. Analyst software was used to evaluate concentrations of the calibration standards, quality controls, and unknowns (version 1.6; Sciex, Darmstadt, Germany). Variations in accuracy, intra-day, and inter-day precision (n = 6 for each concentration, respectively) were <15% over the calibration range. The lower limits of quantification were 0.1 ng/mL for anandamide, 0.25 ng/mL for 2-AG, and 0.5 ng/mL for PEA and OEA.

#### 2.5.2 Analysis of lysophosphatidic acids

Sample extraction was performed with liquid–liquid extraction as already described (13). Therefore, 50 µL plasma was extracted twice with 500 µL of water-saturated n-butanol. The LC-MS/MS system was the same as described for endocannabinoids. For the chromatographic separation, a Luna C18 (14) Mercury column was used (20 x 2 mm inner diameter, 5 µm particle size, and 100 Å pore size, Phenomenex, Aschaffenburg, Germany) with the same material precolumn. A linear gradient was run at a flow rate of 0.4 mL/min for the separation of the analytes with a total run time of 7 minutes. Mobile phase A was 50 mM ammonium acetate containing 0.2% formic acid, and mobile phase B was acetonitrile:isopropyl alcohol:formic acid (50:50:0.2, v/v/v). Quantification was performed using the internal standard method with Analyst software version 1.5 (Sciex, Darmstadt, Germany). Ratios of analyte peak area and internal standard area (*y*-axis) were plotted against concentration (*x*-axis), and calibration curves were calculated by linear regression with 1/x concentration weighting. The coefficient of correlation was at least 0.99. Variations in accuracy were less than 15% over the range of calibration.

#### 2.5.3 Analysis of eicosanoids

The following lipid mediators were analyzed using liquid chromatography-tandem-mass spectroscopy (LC-MS/MS): Leukotriene B4 (LTB_4_); hydroxyeicosatetraenoic acids 5(S)-HETE, 12(S)-HETE, 15(S)-HETE and 20(S)-HETE); epoxyeicosatetraenoic; and dihydroxyeicosatrienoic acids (5,6-DHET; 8,9-DHET; 11,12-DHET;14,15-DHET). The LC-MS/MS system consisted of a 5500 QTrap mass spectrometer (Sciex, Darmstadt, Germany), operating in negative ESI mode, an Agilent 1200 HPLC system (Agilent, Waldbronn, Germany), and an HTC Pal autosampler (Chromtech, Idstein, Germany).

Sample extraction of LTB_4_, HETEs, EETs, and DHETs was performed using liquid–liquid extraction: 200 µL of plasma were gently mixed with 20 µL of methanol and 20 µL of internal standard solution and extracted twice with 600 µL ethyl acetate. Samples for standard curve and quality control were prepared similarly: 200 µL PBS, 20 µL of standard solution, and 20µL internal standard solution were mixed and extracted with ethyl acetate. Working solutions of all analytes were prepared in methanol containing 0.1% BHT. The calibration standards were prepared by further dilution of the working standards.

The organic phase was removed at 45°C under a gentle stream of nitrogen. The residues were reconstituted in 50 µL of methanol:water:BHT (50:50:10–^4^, v/v/v) prior to injection into the LC-MS/MS system. Chromatographic separation was achieved using a Gemini NX C18 column (150 mm × 2 mm ID, 5 µm, Phenomenex, Aschaffenburg, Germany) with a pre-column of the same material. A linear gradient was employed at a flow rate of 0.5 mL/min and a total run time of 17.5 minutes. Mobile phases were A water:ammonia (100:0.05, v/v) and B acetonitrile:ammonia (100:0.05, v/v). The gradient started at 85% A, changed to 10% A within 12 min, held for one min, and shifted back to 85% A in 0.5 min following 3.5 min equilibration.

All data were acquired using Analyst software v1.6.2, and quantitation was performed by MultiQuant software v3.0 (both Sciex, Darmstadt, Germany) using the internal standard method (isotope-dilution mass spectrometry). Calibration curves were calculated by linear regression with 1/x or 1/x^2^ weighting, and acceptance criteria were applied as described previously (15).

Prostaglandins sample analysis was performed using liquid chromatography-electrospray ionization-tandem mass spectrometry (LC-ESI-MS/MS) as described elsewhere (16). The LC-MS/MS system was the same as for endocannabinoid analysis. For the chromatographic separation, a Synergi Hydro-RP column and pre-column were used (150 x 2 mm ID, from Phenomenex, Aschaffenburg, Germany). A linear gradient was employed at a flow rate of 300 µL/min. Mobile phase A was water:formic acid (100:0.0025, v/v, pH 4.0) and mobile phase B was acetonitrile:formic acid (100:0.0025, v/v). The sample solvent was acetonitrile:water:formic acid (20:80:0.0025, v/v, pH 4.0). The total run time was 16 minutes, and the injection volume of samples was 20 µL. Quantitation was performed with Analyst software V1.5 (Sciex, Darmstadt, Germany) using the internal standard method (isotope-dilution mass spectrometry). Ratios of analyte peak area and internal standard peak area (y-axis) were plotted against concentration (x-axis), and calibration curves for each prostaglandin (PGE_2_, PGD_2_, PGF_2alpha_) were calculated by least square regression with 1/concentration^2^ weighting.

#### 2.5.4 Analysis of linoleic acid derivatives

Lipid identification and quantification were performed as previously described (17). Briefly, stock solutions with analytes: 9-hydroxyoctadecadienoic acid (9-HODE), 13-HODE, 9,10-epoxy-12Z-octadecenoic acid (9,10-EpOME) 12,13-EpOME, 9,10-dihydroxy-12Z-octadecenoic acid (9,10-DiHOME), 12,13-DiHOME, and the corresponding internal standards were prepared in methanol. Sample pretreatment and LC-MS/MS analysis were performed as described in Section 2.5.3.

#### 2.5.5 Analysis of sphingolipids

For lipid extraction, 10 µL of plasma was mixed with 150 µL water, 150 µL extraction solution (citric acid 30 mM, disodium hydrogen phosphate 40 mM), and 20 µL internal standard solution as described elsewhere (18). Afterward, the amounts of sphingolipids were analyzed by liquid LC-MS/MS. An Agilent 1100 series binary pump (Agilent Technologies, Waldbronn, Germany) equipped with a Luna C8 column (150 mm x 2 mm ID, 3µm particle size, 100 Å pore size; Phenomenex, Aschaffenburg, Germany) was used for chromatographic separation. The column temperature was set at 35°C. The HPLC mobile phases consisted of water with 0.2% formic acid and 2 mM ammonium formate (mobile phase A) and acetonitrile:isopropanol:acetone (50:30:20, v/v/v) with 0.2% formic acid (mobile phase B). For separation, a gradient program was used at a flow rate of 0.3 mL/min. The MS/MS analysis was performed using a triple quadrupole mass spectrometer API4000 (Sciex, Darmstadt, Germany) equipped with a Turbo V Ion Source operating in positive electrospray ionization mode. Data acquisition was performed using Analyst software V 1.6, and quantification was performed with MultiQuant software V 3.0 (both Sciex, Darmstadt, Germany), employing the internal standard method (isotope-dilution mass spectrometry). Variations in the accuracy of the calibration standards were less than 15% over the whole calibration range, except for the lower limit of quantification, where a variation in accuracy of 20% was accepted. Using this method, we analyzed plasma levels of sphingolipids listed in **Table 3**.

### 2.6 Statistical analysis of all lipids in healthy volunteers and ICU patients

All statistical analysis, including tests and graphs of the final data set (experimental values obtained from the laboratory analysis), was made using Graphpad Prism (version 9.5.0) and R software (version 3.5.1, open-source software 2018). The distribution of variables was pre-tested for normality using the Shapiro-Wilk-test to decide which kind of tests to apply later on (t-test or Wilcoxon-Mann-Whitney) and to test indirectly for symmetry to decide which kinds of location parameter (mean ± x-fold standard deviation or median ± upper and lower 95% range limits) should be taken for the descriptive analysis. For lipid plasma levels, the respective normal ranges were then defined as the median ± upper and lower normal range limit (NRL_95%_), indicating the range of lipid plasma levels shown by at least 95% of healthy humans. We used the median instead the mean, as the samples were asymmetric and not normally distributed. We calculated the 95% limit instead of quartiles because clinical laboratory plasma values are usually given as threshold ranges (normal or reference ranges) representing the plasma levels found in 95% of healthy subjects. The normal ranges for the classical lipids IL-6 and leukocytes were adopted from the reference ranges of Frankfurt University Hospital’s central clinical laboratory. For the lipids, we used as references the plasma sample of healthy volunteers (see sections 2.1, 2.3, and 2.4). We defined “significantly out of normal range” if plasma levels are outside the area defined by median_NR_ ± 2-fold upper and lower limit NRL_95%_. Lipids with at least one plasma peak level outside the range median_NR_ ± 2-fold upper and lower limit NRL_95%_, observed in at least five of the nine patients, were classified as “prevalently regulated”.

Lipid plasma levels were illustrated with time-dependent graphs expressed as hours stayed at the ICU, starting at zero (day one after surgery. The NRL_95%_ normal range is indicated by dotted lines. The range median_NR_ ± 2-fold upper and lower limit NRL_95%_ is shown as gray background. In few cases, where lipid plasma levels measurement errors are suspected, the data points are not included in the curves but are displayed as single data points for transparency reasons.

For the *ex vivo* whole blood LPS assay, the pairwise Wilcoxon test (two-sample test for dependent, non-normally distributed samples) was used because several samples demonstrated an explicit deviation from normal distribution, while for others, there was a lack of sufficient data points for distribution analysis. Unstimulated controls were compared with samples that had been treated. “Significantly regulated” was used if at least one incubation period with LPS (6 h, 16 h, 24 h) versus the control was significantly different (p < 0.05). Due to the study design, no significance correction for multiple testing was applied. This was deliberate, as such a procedure would have changed the results negligibly.

## 3 Results

### 3.1 Clinical course of the patients included after cardiac surgery

The average stay at the ICU was 43.2 days (min 8 days, max. 101 days). All patients received high dosages of vasopressors and inotropics at the beginning of intensive care therapy. The average SOFA score (modified SOFA using the Richmond Agitation-Sedation Scale in critically ill patients according to (19)) was 11.7 (min 8, max 15) at admission. There were four non-survivors (**Table 1**).

### 3.2 *In vivo* lipid mediator profile in ICU patients after cardiac surgery

**Figure 2:**
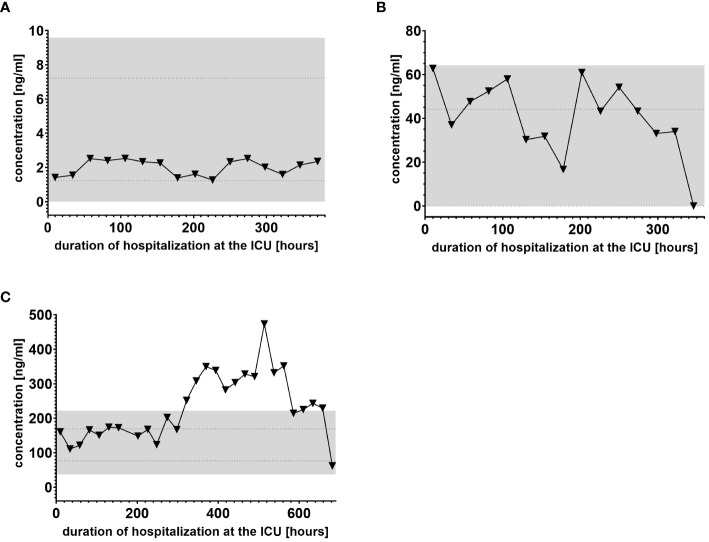
Examples of different lipid mediator reactivity during clinical inflammatory stages. **(A)** Example of a lipid mediator with plasma levels within the normal range limit of NRL_95%_ and therefore considered “not regulated.” **(B)** Example of lipid mediator regulated just above the normal range limit of NRL_95%_ and therefore classified as “regulated.” **(C)** Example of a lipid mediator with plasma levels outside the gray-colored 2-fold NRL_95%_ area and consequently classified as “significantly regulated.” Graphs of the final data set were created using Graphpad Prism version 9.5.0.

In the present study we analyzed baseline values of all lipids investigated in this study since their normal ranges in human plasma are largely unknown. For ceramides, normal ranges based on plasma levels of healthy subjects were similar to those reported in literature. E.g. baseline ceramide level in our study are consistent with those published by others (i.e. Cer (d18:1/16:0): 0.220 ± 0.044 µM *vs* 0.24–0.30, Cer (d18:1/18:0): 0.067 ± 0.028 µM *vs* 0.001–0.11; Cer (d18:1/24:0): 4.490 ± 1.435 µM *vs* 1.15–3.34; Cer (d18:1/24:1): 0.746 ± 0.240 µM *vs* 0.71–1.14 (20–23). Comparisons of baseline values of other lipids investigated in this study could not be established, since their normal ranges in human plasma are largely unknown. **Figure 2** shows examples of different lipid reactivity during the stages of systemic inflammation. Notably, the type of lipid is not mentioned at this stage, as the purpose of this figure is to introduce different types of lipid mediator reactivity with respect to normal ranges. **Figure 2A** shows an example of an unchanged lipid mediator. **Figure 2B** shows an example of a lipid mediator changed just above the normal range but still below the medianNR ± 2-fold upper and lower limit NRL95%. **Figure 2C** shows an example of a significantly regulated lipid mediator. Lipids that displayed plasma levels remaining consistently within the normal range during hospitalization, such as that shown in **Figure 2A**, were not evaluated further. We identified 22 of the 53 lipids which showed significant regulation during at least one time point on the ICU ward among at least 5 out of 9 patients, thus indicating potential clinical relevance. This group of prevalently regulated lipid mediators with potential clinical relevance could be further divided into lipids with either down-or upregulated levels during these general inflammatory conditions on ICU (**Table 4**).

**Table 4:** Lipid mediators of the ICU patient cohort (n = 9) compared with the effect of LPS stimulation on the plasma levels of selected lipids of healthy volunteers (n = 10) analyzed by LC-MS/MS.

Lipid	Significant effect in the LPS assay	Prevalent effects in ICU patients	Effect similarity LPS-assay vs. patients
Cer (d18:0/16:0)	↑	●	No
Cer (d18:0/18:0)	↑	↑	Yes
Cer (d18:0/24:0)	●	●	Yes
Cer (d18:1/14:0)	●	●	Yes
Cer (d18:1/16:0)	↑	↑	Yes
Cer (d18:1/18:0)	↑	●	No
Cer (d18:1/18:1)	↑	↑	Yes
Cer (d18:1/20:0)	↑	●	No
Cer (d18:1/24:0)	↓	↓	Yes
Cer (d18:1/24:1)	●	●	Yes
Cer (d18:0/24:1)	●	●	Yes
GlcCer (d18:1/16:0)	●	●	Yes
GlcCer (d18:1/18:0)	●	●	Yes
GlcCer (d18:1/24:1)	●	↑	No
LacCer (d18:1/16:0)	↑	↓	No
LacCer (d18:1/18:0)	↑	↑	Yes
LacCer (d18:1/24:0)	↑	↓	No
LacCer (d18:1/24:1)	↑	↑	Yes
SPH d18:0	↑	●	No
S1P d18:0	↓	↓	Yes
SPH d18:1	↑	↑	Yes
S1P d18:1	↓	↓	Yes
LTB_4_	—	●	—
PGE_2_	↑	●	No
PGD_2_	↑	●	No
PGF_2_alpha	↑	●	No
6-keto-prostaglandin F1alpha	●	↑	No
Thromboxane B2	●	●	Yes
5-HETE	—	●	—
12-HETE	—	●	—
15-HETE	—	●	—
20-HETE	—	↑	—
5,6 DHET	—	●	—
8,9 DHET	—	●	—
11,12 DHET	—	↑	—
14,15 DHET	—	↑	—
AEA	↑	●	No
OEA	↑	●	No
PEA	↑	●	No
1-AG	●	↑	No
2-AG	●	↑	No
LPA (16:0)	↑	↓	No
LPA (18:0)	↑	↓	No
LPA (18:1)	↑	↓	No
LPA (18:2)	↑	●	No
LPA (18:3)	↑	●	No
LPA (20:4)	↑	●	No
9,10 EpOME	—	●	—
12,13 EpOME	—	●	—
9,10 DiHOME	—	●	—
12,13 DiHOME	—	●	—
9-HODE	—	↓	—
13-HODE	—	●	—

“Prevalently regulated” in patients was used if the lipid was significantly regulated out of normal range during at least one point in the ICU stay in at least five of the nine patients, representing the majority of subjects. “Significantly regulated” in the LPS assays was used if at least one incubation period with LPS (6 h, 16 h, 24 h) versus the control was significantly different (p < 0.05). For further details on the statistical analysis see section 2.6. ↑ = lipid significantly upregulated, ↓= lipid significantly downregulated, ●= lipid not regulated, — = no data available.

**Figure 3:**
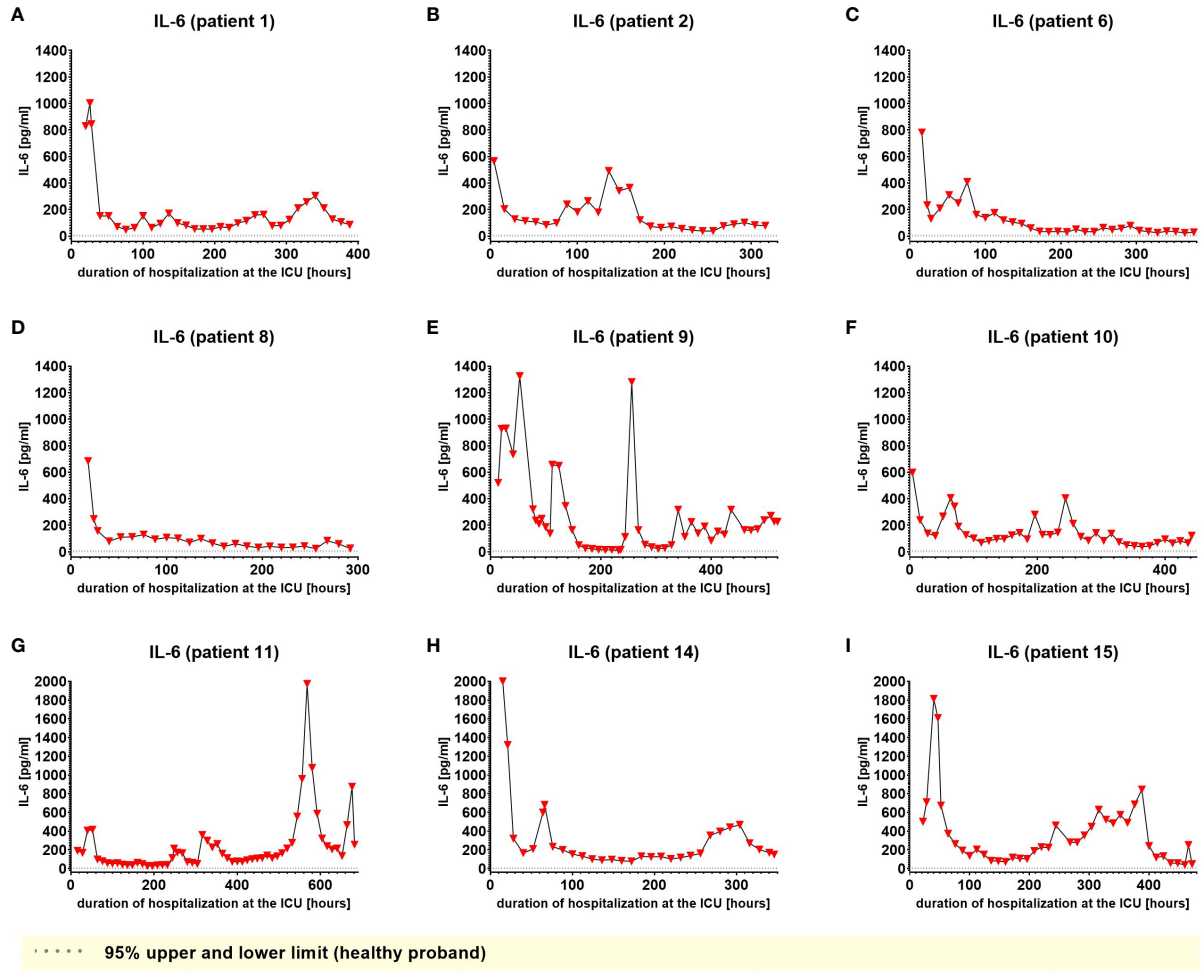
Plasma levels of IL-6 in nine hospitalized patients at the ICU analyzed at different times in different patients **(A–I)** using LC-MS/MS as described in material and methods. The normal ranges are visualized as dotted lines. Graphs of the final data set were created using Graphpad Prism (version 9.5.0).

**Figure 4:**
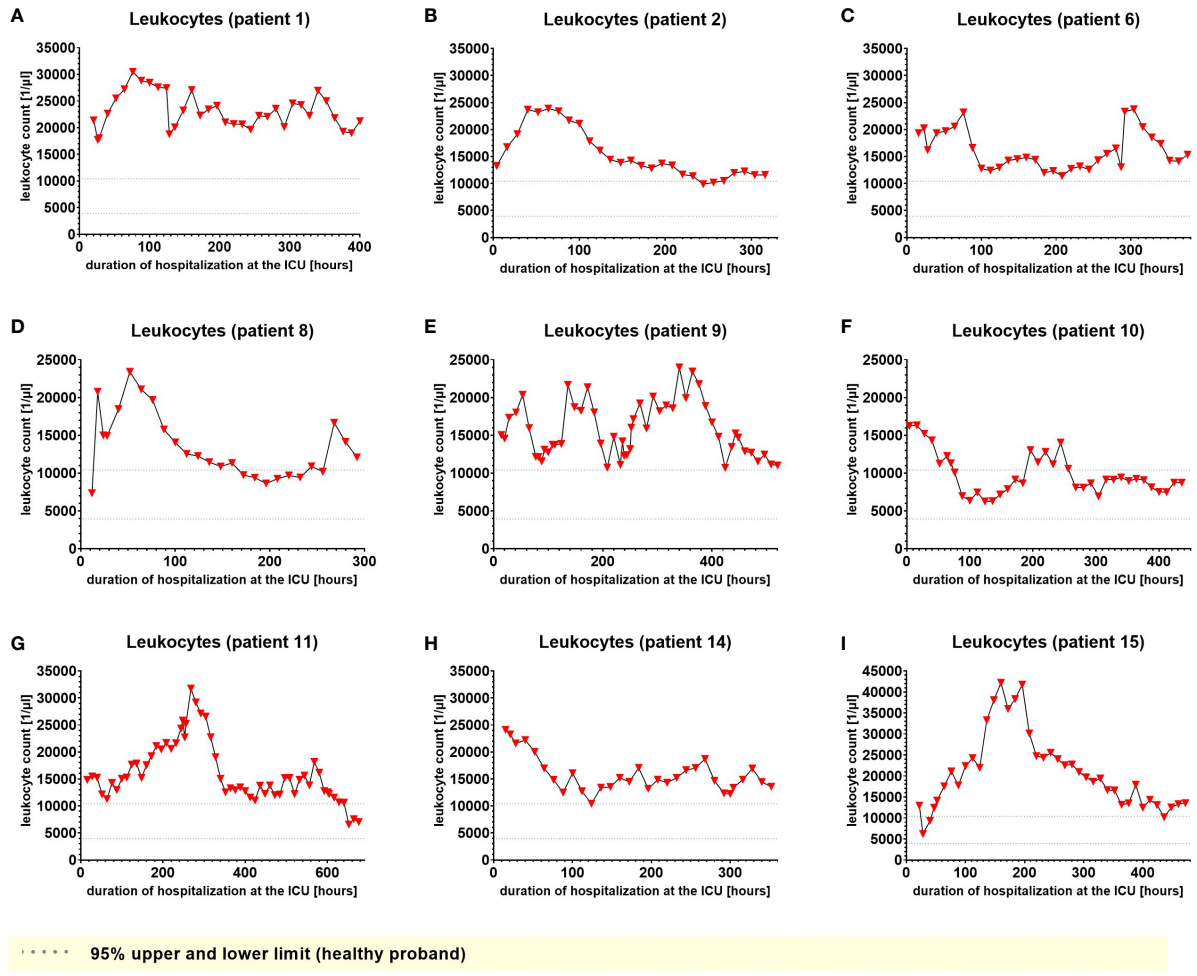
Leukocyte count in EDTA-stabilized venous whole blood of nine hospitalized patients at the ICU analyzed at different times in different patients **(A–I)** using LC-MS/MS as described in material and methods. The normal range is presented as dotted lines. Graphs of the final data set were created using Graphpad Prism (version 9.5.0).

**Figure 5:**
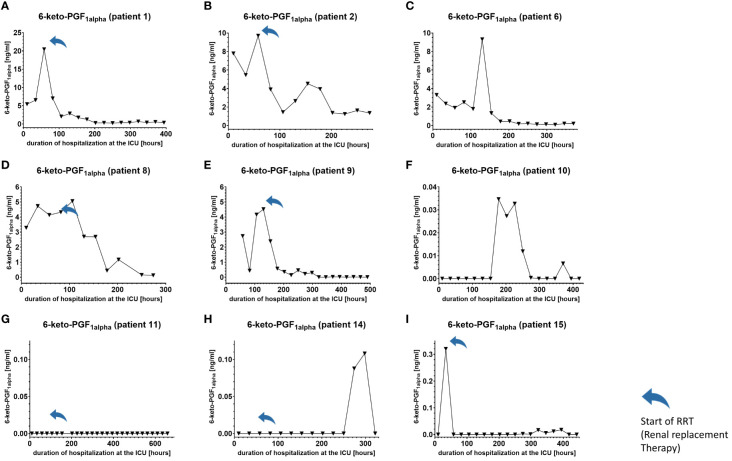
Plasma levels of 6-keto-PGF1_alpha_ in nine hospitalized patients at the ICU analyzed at different times in different patients **(A–I)** using LC-MS/MS as described in the Material and Methods section. The normal range is visualized as dotted lines and the range median ± 2-fold upper and lower limit NRL_95%._ as gray background. Notably, normal range 6-keto-PGF1_alpha_ levels were below the lower limit of quantification of LC/MS-MS analysis and are therefore not visible in the figure. Graphs of the final data set were created using Graphpad Prism (version 9.5.0).

**Figure 6:**
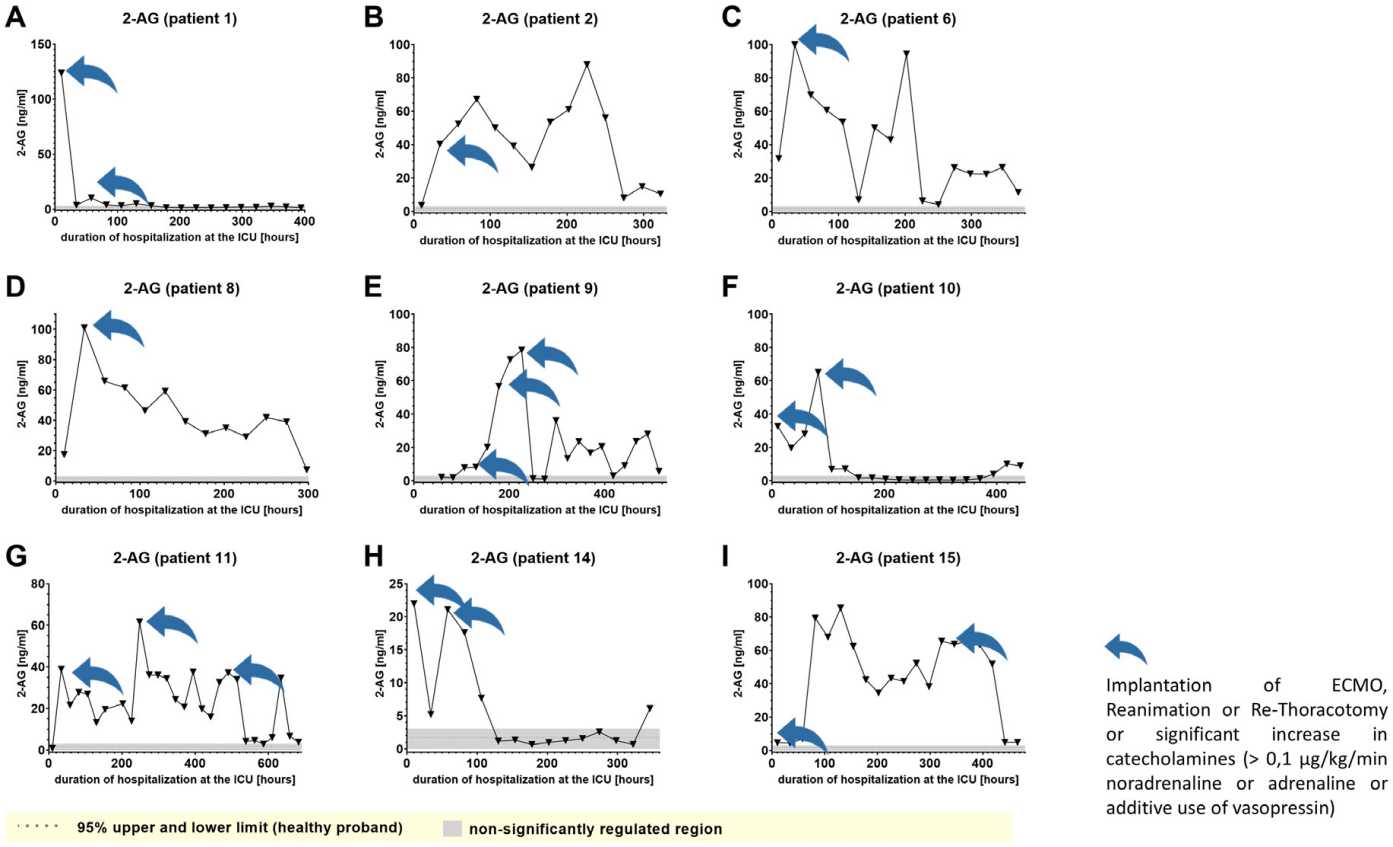
Plasma levels of 2-AG in nine hospitalized patients at the ICU analyzed at different times in different patients **(A–I)** using LC-MS/MS as described in material and methods. The normal range of lipids is visualized as dotted lines, and the range median ± 2-fold upper and lower limit NRL_95%._ as gray background. Graphs of the final data set were created Graphpad Prism (version 9.5.0).

We used IL-6 and leukocyte count as established markers of inflammation (**Figures 3**, **4**). Interestingly, the leukocyte count remained consistently elevated in most patients, pointing to the severity of illness the patients were suffering from, rather than specific clinical events. The relevant lipid plasma peaks were then compared with clinical phases. Regarding the type of plasma level regulation during the observation period and clinical events, we were able to define two major groups of lipids. The first group (dynamically upregulated lipids) features plasma levels rapidly changing from the normal range to the area above the twofold normal range upper NRL95%-limit. Based on visual interpretation for some of these lipids, changes in plasma levels seemed to have limited correlation with patients’ inflammatory status. **Figures 5** and **6** show representative graphs for 6-keto-PGF1_alpha_ and 2-AG. These two lipids partly correlated with inflammatory stages but rather seemed to correlate more to some specific clinical events. While 6-keto-PGF1_alpha_ had its peak shortly before initiation of continuous veno-venous hemodialysis (at least in 5 of the observed patients, see **Figure 5**), 2-AG peaked in all our nine patients during (right) heart failure in the context of either re-opening patient’s chest, implementation of veno-arterial ECMO or at least while significantly increasing the amount of catecholamines (see **Figure 6**). Besides these visual correlations, the remarkable increase of 6-keto-PGF1alpha in certain patients becomes more noteworthy considering that normal range levels of this lipid in healthy volunteers are below the detection limit of LC/MS-MS.

**Figure 7:**
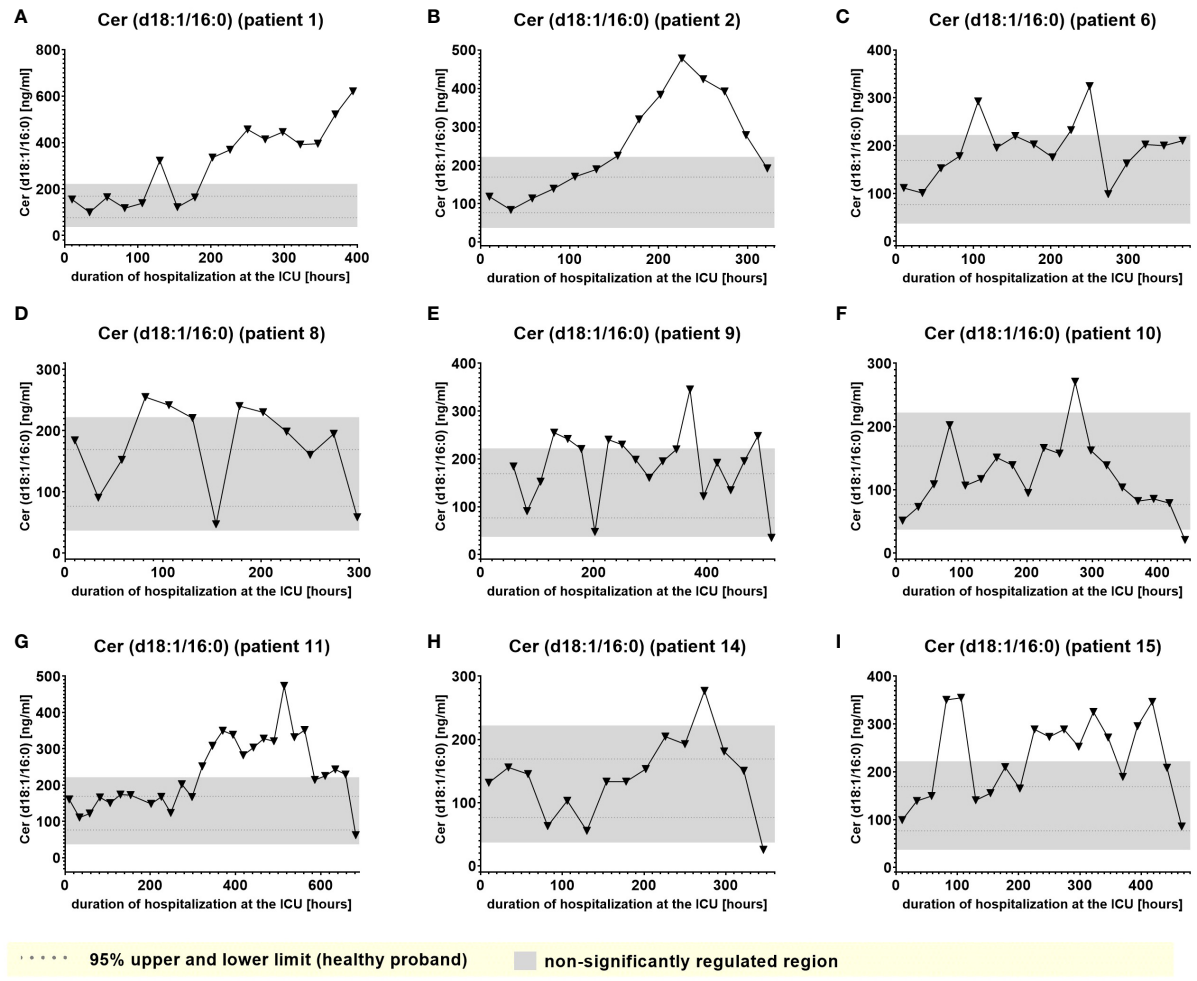
Plasma levels of Cer (d18:1/16:0) in nine hospitalized patients at the ICU analyzed at different times in different patients **(A–I)** using LC-MS/MS as described in material and methods. The normal range is visualized as dotted lines, and the range median ± 2-fold upper and lower limit NRL_95%._ as gray background. Graphs of the final data set were created Graphpad Prism (version 9.5.0).

Furthermore, Cer (d18:1/16:0), Cer (d18:1/18:1) and LacCer (d18:1/24:1) showed a tendency to accumulate in the plasma during the time of hospitalization and remained elevated for prolonged periods (**Figure 7** and **Supplementary Figures 2A–1**, **5A–I**). Notably, 6-keto-PGF1_alpha_ 1-AG, 2-AG and 20-HETE were the lipids that displayed the strongest maximal relative induction of biosynthesis compared with the plasma normal range concentrations in healthy persons. Among these, the strongest relative induction was seen with 6-keto-PGF1_alpha_ levels reaching up to 20 ng/ml and basal levels below the detection limit, followed by about 350-fold (2-AG), 200-fold (1-AG) and 40-fold maximal induction (20-HETE).

**Figure 8:**
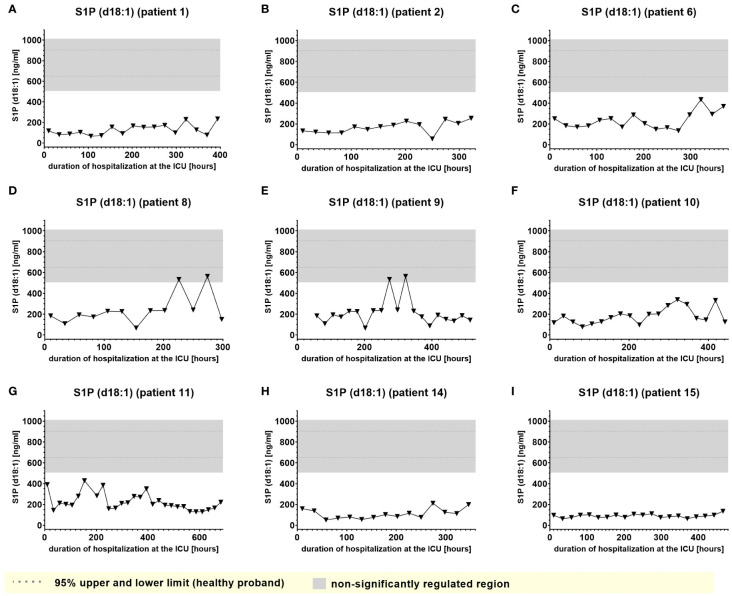
Plasma levels of S1P d18:1 in nine hospitalized patients at the ICU analyzed at different times in different patients **(A–I)** using LC-MS/MS as described in material and methods. The normal range of the lipids is visualized as dotted lines, and the range median ± 2-fold upper and lower limit NRL_95%_ as gray background. Graphs of the final data set were created using Graphpad Prism (version 9.5.0).

**Figure 9:**
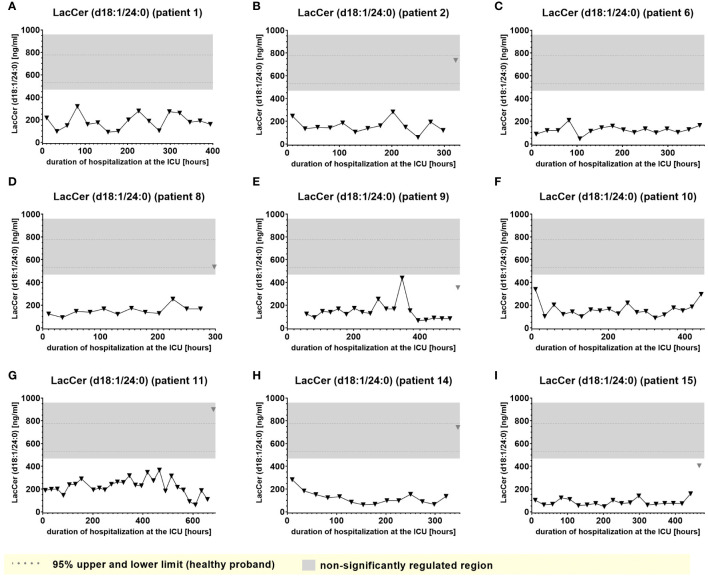
Plasma levels of LacCer (d18:1/24:0) in nine hospitalized patients at the ICU analyzed at different times in different patients **(A–I)** using LC-MS/MS as described in material and methods. The normal range of the lipids is visualized as dotted lines, and the range median ± 2-fold upper and lower limit NRL95% as gray background. Graphs of the final data set were created using Graphpad Prism (version 9.5.0).

A second group of lipids (significantly downregulated lipids) demonstrated plasma levels significantly below the normal range that, however remained impaired consistently and to similar degree during almost the entire time of hospitalization and were largely irrespective of clinical events. Examples of this type of lipid mediator, such as plasma levels of S1P (d18:1) and Lactosylceramide LacCer (d18:1/24:0), are shown in **Figures 8** and **9**.

Several lipids displayed rather low normal-range concentrations, with lower NRL95% limits already close to the LC-MS/MS detection limit and levels that apparently were further reduced during hospitalization. This applies for example to LPA (18:2), LPA (18:3), LPA (20:4), 9,10-DiHOME, PEA, OEA and PGE_2_. Surprisingly, we did not observe any relevant elevation of the classical pro-inflammatory products of the cyclooxygenase-2 and 5-lipoxygenase pathway, including PGE_2_ and 5-HETE or LTB_4_. All graphs of prevalently regulated lipids with potential clinical relevance (see **Table 4**) not shown in **Figures 5**–**9** can be found in the supplemental section, **Supplementary Figures 1**-**17**.

### 3.3 *Ex vivo* lipid mediator profile in LPS-stimulated human whole blood

Next, we compared the changes of lipid mediators from patients with systemic inflammation with an *ex vivo* model of endotoxin-induced inflammation. Here, venous whole blood from healthy volunteers was stimulated with LPS for 6 h, 16 h, and 24 h. The lipid mediator profile included 38 lipids analyzed using LC-MS/MS. Notably, due to a technical failure during LC-MS/MS analysis, only 38 of the 53 lipid mediators could be analyzed, however including all important lipids in patients described above.

**Figure 10:**
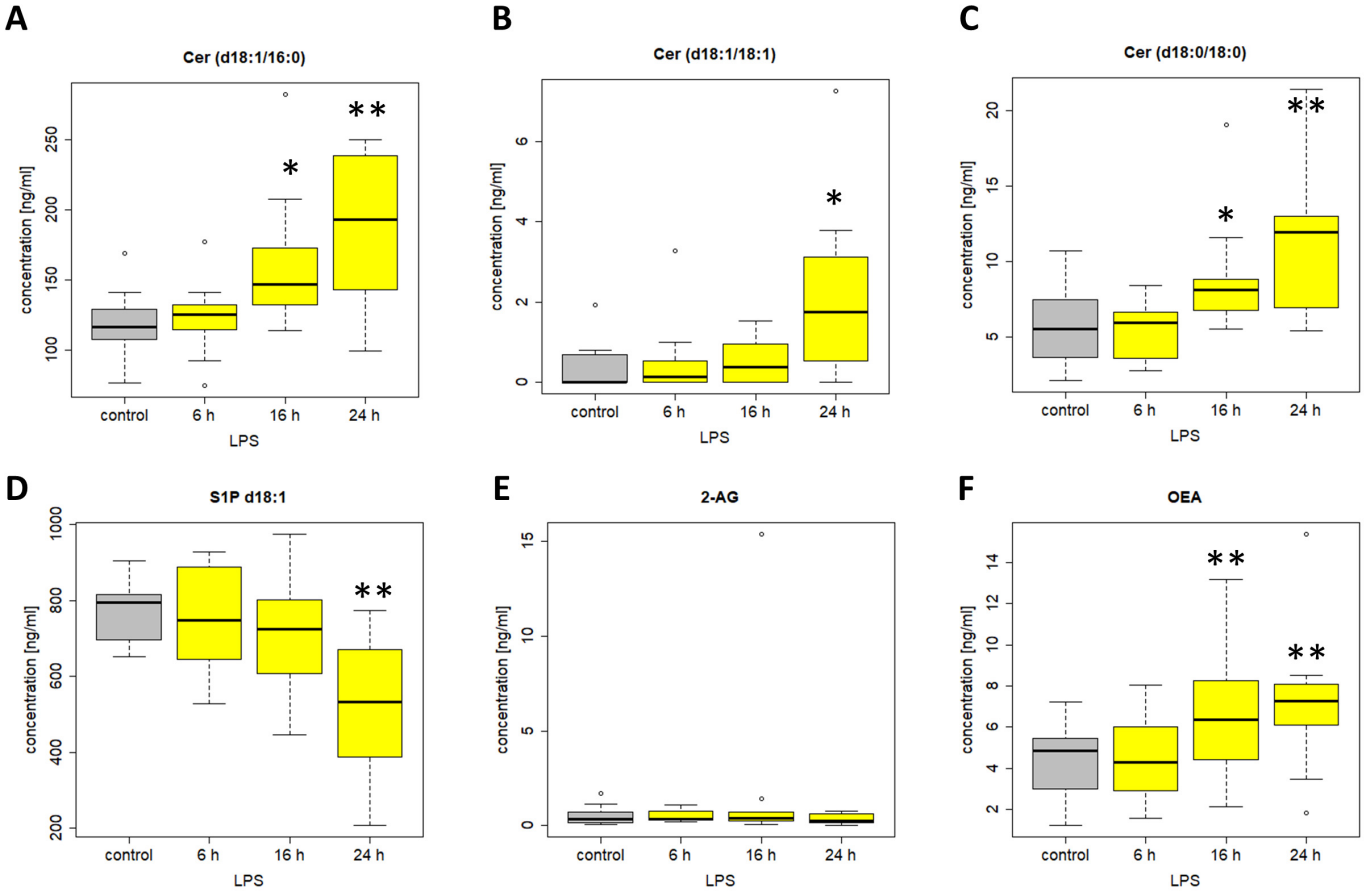
Plasma levels of **(A)** Cer (d18:1/16:0) **(B)** Cer (d18:1/18:1) **(C)** Cer (d18:0/18:0) **(D)** S1P d18:1 **(E)** 2-AG **(F)** OEA after stimulation of venous whole blood with 100 ng/ml LPS for the time periods indicated. Samples were further handled as described in material and methods and plasma levels were determined using LC/MS/MS. Data are median of 10 independent experiments illustrated as box plots with upper and lower whiskers. LPS, lipopolysaccharide. Levels of significance of control versus sample treated with LPS: *p< 0.05; **p<0.01. Graphs and statistics of the final data set were created using R software (version 3.5.1, open-source software 2018).

**Figure 11:**
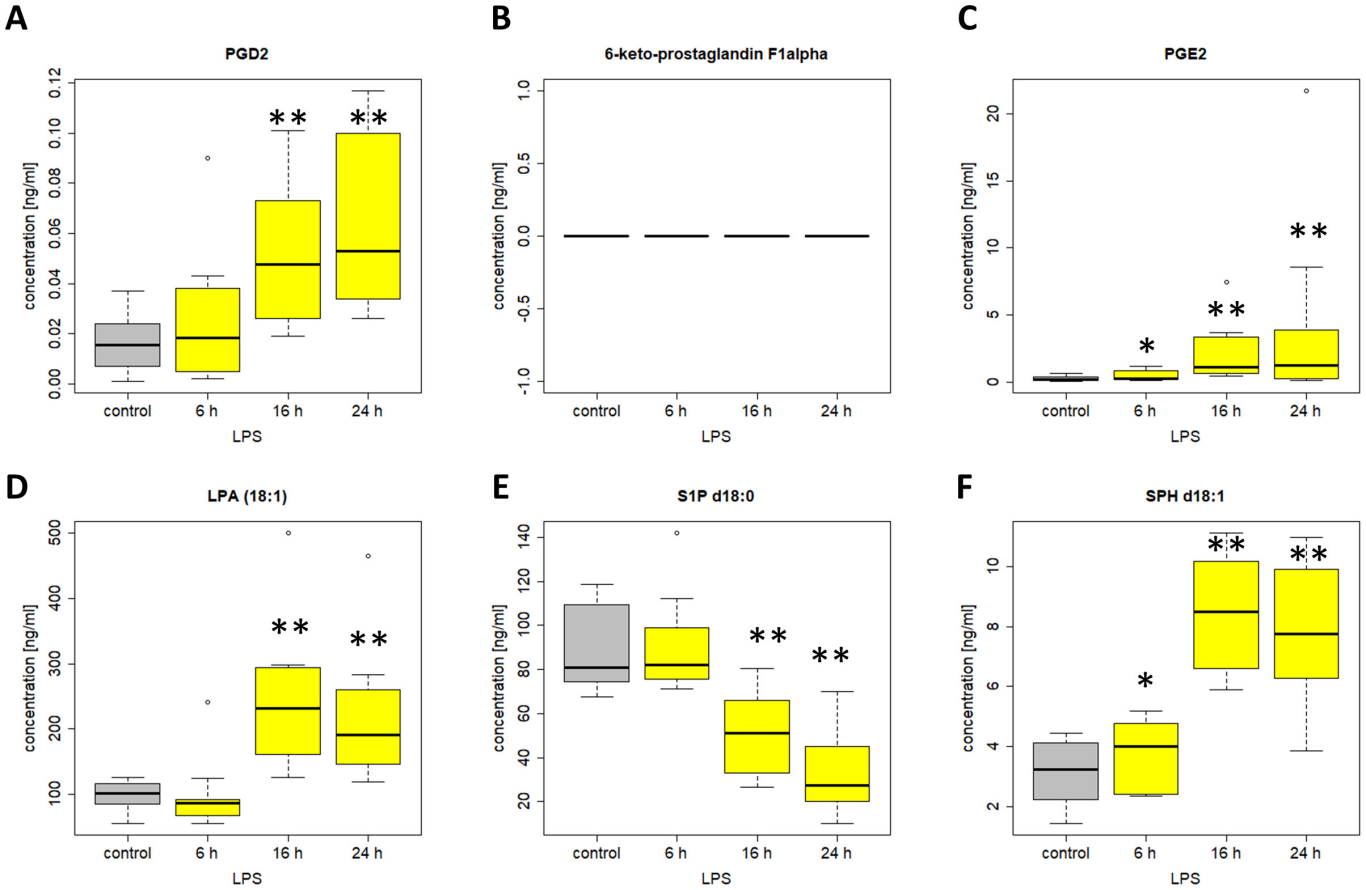
Plasma levels of **(A)** PGD2 **(B)** 6-keto-PGF1alpha **(C)** PGE2 **(D)** LPA 18:1 **(E)** S1P d18:0 **(F)** SPH d18:1 after stimulation of venous whole blood with 100 ng/ml LPS for the time periods indicated. Samples were further handled as described in material and methods and plasma levels were determined using LC/MS/MS. Data are median of ten independent experiments illustrated as box plots with upper and lower whiskers. LPS, lipopolysaccharide. Levels of significance of control versus sample treated with LPS: *p< 0.05; **p<0.01. Graphs and statistics of the final data set were created using R software (version 3.5.1, open-source software 2018.

Among the 38 included lipids we identified 27 lipid mediators with significantly changed plasma levels after incubation with LPS, as compared to the untreated control. **Figures 10** and **11** include graphs for selected lipids from this LPS-based assay that either had already shown significant regulation in the ICU patients or only showed reactivity in our LPS model but not in our patients. Graphs of lipid plasma levels after stimulation of whole blood with LPS not included in **Figures 10** and **11** can be found in **Supplementary Figures 18**-**22**. Further details on the results of the LPS analysis at different time points can be found in **Supplementary Table 1**. The earliest significant increase compared with the control after 6 hours was seen with prostaglandin PGE_2_ (**Figure 11C**), PGF_2alpha_ and Cer (d18:0/16:0) (**Supplementary Figures 18A, 20E**) followed by PGD_2_ (**Figure 11A**), although these lipids were not regulated in our patients. The early and significant induction of classical pro-inflammatory mediators such as PGE_2_ confirmed successful stimulation by LPS. Further significantly increased plasma level elevations were seen after 16 hours for several lipids that in most cases had shown strong equally directed regulation in our patients. Such similar effects were observed with the majority of ceramides. These lipids remained significantly elevated after 24 hours in the majority of cases. A late but significant increase after 24 hours was observed for Cer (d18:1/18:1) (**Figure 10B**). As observed in our patients, three lipids were significantly downregulated in the whole blood assays after LPS treatment, including S1P (d18:1) (Sphingosin-1-phosphat), Cer (d18:1/24:0), and S1P (d18:0) (Sphinganine-1-Phosphat) (**Figures 10D**, **11E**, **Supplementary Figure 18F**). No effect was seen using the LPS assay for GlcCer (d18:1/24:1), 1-AG, 2-AG, and 6-keto-PGF1alpha, although these lipids were strongly regulated in our patients. Finally, all endocannabinoids and all lysophosphatidic acids displayed opposite changes in plasma levels in patients and the whole blood assay. Of the 38 lipids investigated, only 16 showed a similar reactivity both in our patients and in our *ex vivo* model of systemic inflammation. **Table 4** summarizes the effect of LPS on the biosynthesis of all lipid mediators in whole blood, including information on a possible regulation in patients and information on a possible similar reactivity under both conditions.

## 4 Discussion

Eicosanoids, sphingolipids, and endocannabinoids belong to the group of bioactive lipids crucially involved in several inflammatory diseases such as bronchial asthma, local inflammation, allergy, and arthritis (24). However, their role in systemic inflammatory responses in critically ill patients is only partly understood. We initially hypothesized that plasma levels of at least some lipids might be changed during e.g. sepsis periods, theoretically providing a possible rationale for their use as new biomarkers or for identifying crucial signaling pathways and new therapeutic strategies. Therefore, plasma levels of several lipids from nine ICU patients following cardiac surgery were analyzed daily during hospitalization and compared with clinically established biomarkers of inflammation such as leukocytes and IL-6. We found that 22 of the 53 lipids analyzed by LC-MS/MS were significantly changed during clinical events. Among these, 2 lipid mediators showed a possible increase during acute kidney injury or (right) heart failure respectively, while the established inflammation parameters such as IL-6 only showed a congruency to a low degree with 11 other lipid mediators. Another group of 9 lipids displayed plasma levels that were consistently lower than the normal range.

Several studies investigating the role of lipids in sepsis have confirmed the crucial role of certain lipids such as ceramides, PGE_2_, LTB_4_ and S1P (d18:1) in the pathogenesis of this severe immune dysregulation (1–4). However, to our knowledge, no previous study has analyzed such a large number of different lipid levels using a highly sensitive LC/MS-MS methodology at that many time points throughout the patients stay at the ICU. Moreover, our study reflects lipid levels in hospitalized patients under clinically realistic conditions rather than using standardized and simplified animal models of disease or even *ex vivo* or *in vitro* models of sterile inflammation.

Some studies investigated the role of eicosanoids and sphingolipids in sepsis. The role of elevated levels of certain cyclooxygenase-2-derived prostanoids with effects on the hemodynamic and inflammatory processes during sepsis has been published (25–27). Accordingly, we could confirm these findings by showing that the COX-2-derived metabolite 6-keto-PGF_1alpha_ is elevated in almost all patients. 6-keto-PGF_1alpha_ is a stable hydrolysis product of prostacyclin (PGI_2_). Given the short half-life of PGI2 in plasma (a few minutes), its synthesis is more accurately reflected by measurement of the 6-keto-PGF_1alpha_ metabolite. PGI_2_ has several functions that include the promotion of platelet aggregation and thrombosis, triggering endothelial adhesion and extravasation of neutrophils at sites of inflammation, and suppressing vasoconstrictor responses to pressor hormones, thereby crucially contributing to the clinical appearance of sepsis (28) and as observed here, acute kidney injury. The increased use of vasopressors and the subsequent vasopressor desensitization might play a significant role in our observed patients. Anyhow, our study attaches importance to the pathophysiological role of PGI_2_ and strongly encourages further research into the role of this lipid and 6-keto-PGF_1alpha_.

In accordance with a study by Bruegel et al., we did not observe any relevant elevation of prostaglandin E_2_ or 5-lipoxygenase pathway products (e.g. 5-HETE or LTB_4_) in the patients, particularly as these synthesis pathways are suppressed under septic conditions (5). Increased levels of Cer (d18:1/16:0), Cer (d18:1/18:1) and Cer (d18:0/18:0) were tracked in the present study, supporting a number of studies that described increased levels of ceramide in septic patients, which were associated with lower survival rate and poor prognosis (29). This aligns well with recent studies suggesting that ceramides are pathophysiologic relevant players in cardiovascular diseases involved in regulation of blood pressure, vascular tone and heart function (30). However, Cer (d18:1/16:0), and to a lower degree, Cer (d18:1/18:1), displayed a tendency to accumulate in certain patients, which excludes these metabolites as biomarkers for indicating transitions between clinical stages. On the other hand, such findings raise the important question of their largely unknown potential (patho)physiological role in sepsis. Furthermore, Winkler et al. found inversely decreased S1P (d18:1) concentrations in septic patients. In our study, S1P(d18:1) levels were impaired irrespectively of any clinical stage, excluding S1P(d18:1) as a potential marker to indicate septic stages.

Similar to a previous report by colleagues investigating 2-AG levels in patients during endotoxic shock (31), we found strongly increased concentrations of the endocannabinoids 1- and 2-AG in our patients. The role of endocannabinoids in our setting is largely unclear, as anti-inflammatory and pro-inflammatory effects have been reported (32, 33). We could show that an increase in endocannabinoids is not restricted to the presence of endotoxin and bacterial infections. We can only speculate, how 2-AG levels are linked to (right) heart failure, but it could be an important signaling mediator, causing the typical biological effects of endocannabinoids at cannabinoid receptor 1, such as reduction in anxiety, analgesia, euphoria and impairment of short-term memory (34–36) and also enhancement of hemorrhagic and endotoxin-induced hypotension (37). The increase in 14,15-DHET, which is a direct metabolite and therefore index of 14,15-EET levels, might act as a mechanistic player in the occurrence of any hypotension.

We further demonstrated that LacCer (d18:1/24:0) levels were significantly and consistently reduced in the plasma of the patients included in this study. The limited data suggests that lactosylceramides are more likely to be considered as pro-inflammatory mediators triggering nitric oxide synthesis, oxidative stress and neutrophil activation (38, 39). However, more studies are needed to investigate whether the impaired levels of lactosylceramides might represent an endogenous anti-inflammatory reaction countering an inflammatory process.

For biomarker candidates the degradation/elimination half-life in plasma is of relevance as too long half-life values may prevent the lipid to indicate rapid changes in clinical stages. However, less is known about the half-life of the lipid mediators analyzed in this study. In a study investigating the biosynthetic pathway of very-long and long-chain sphingolipids in 293T cells (40) the turnover rate of different ceramide species could be determined. The authors found that due to their more rapid metabolism into sphingolipids, ceramides containing very-long chain fatty acids such as Cer (d18:1/26:0) and Cer (d18:1/24:1) have a much shorter half-life than long-chain ceramides containing C16:0 and C18:0 fatty acids [Cer (d18:1/16:0), Cer (d18:1/18:1)]. The reported turnover rates for the respective ceramides range from 4,5 h to approximately 24 h for long-chain [i.e. Cer (d18:1/16:0), Cer (d18:1/18:0)] and very-long chain ceramides [i.e. Cer (d18:1/24:0), Cer(d26:1)], respectively. This relatively long half-life may explain persistent plasma level of Cer (d18:1/16:0) and Cer (d18:1/18:1) in certain patients. In contrast to these lipids, the plasma half-life of 1- and 2-AGs and 6-keto-PGF_1alpha_ are in the range of minutes, with the longest reported half-life being 30 min for 6-keto-PGF_1alpha_. Their short half-life may represent an important prerequisite to function as potential biomarker candidates.

Notably, the lipid levels analyzed in the plasma of LPS treated whole blood only partly showed similar alterations compared with changes of lipid plasma levels of the patients in this study. This supports the theory, that *ex vivo* inflammation models such as the LPS assay used in our study only provide rather limited opportunities for predicting changes of lipid mediators at least in critically ill patients.

Retrospectively, some study design settings are, however, limiting the amount and level of information that could be finally obtained from our study. Collecting only one blood sample daily for lipid mediator analysis made it impossible to evaluate the exact rapidity of the plasma lipid mediator level alterations upon transition between clinical stages. Moreover it prevented a comparison with classical biomarkers such as IL-6. Furthermore, the change in lipid plasma levels was partly heterogeneous among the included patients, possibly due to their different medical histories, co-medications, diverse pharmacotherapeutic treatment regimens, and other medical-technical interventions at the ICU such as dialysis, artificial respiration, and extracorporeal life support and other medical-technical interventions at the ICU. Remarkably, the dynamics of lipid mediators may be influenced by norepinephrine infusions affecting the release of certain lipid metabolites (41, 42). In some instances, we observed depleted lipid mediator plasma levels, which might have been due to the well-recognized globally impaired capability of the mediator-synthesizing machinery, such as had been already reported for certain lipids and leukocytes (43). It remains unclear whether there is in fact sufficient “regulatory space” for these lipids to achieve significantly downregulated levels as, for example, non-enzymatic production may also cause a low minimum basal lipid level which cannot be exceeded. Moreover, the same blood collection time interval was applied for all mediators investigated suggesting that possible individual circadian fluctuations in lipid biosynthesis/regulation could not be recorded.

Unfortunately, it was technically impossible to collect subject-related baseline lipid levels prior to surgery and the lipid values of patients therefore had to be compared to normal range of healthy volunteers. However, although a number of parameters potentially disrupted lipid plasma levels of patients, certain lipid mediators, including 6-keto-PGF_1alpha,_ Cer (d18:1/16:0), Cer (d18:1/18:1), 1-AG, 2-AG, and Cer (d18:0/18:0) showed clear and largely consistent changes during the clinical stages in the majority of patients monitored and potentially affect the course of the disease. To partially compensate for these disruptive effects, the level to reach “significant” regulation was therefore elevated in our study (± 2-fold upper and lower limit NRL_95%_). Nevertheless, a number of mediators showed very strong regulation during inflammatory stages highly exceeding these already expanded normal ranges. Regarding the *ex vivo* LPS assay we had to use heparinized blood as the *ex vivo* biosynthesis of a number of lipids is dependent on calcium influx into immune cells making the use of citrated blood impossible because of calcium depletion. Thus, lipid biosynthesis after the ex vivo stimulation of whole blood using LPS may not fully reflect the situation in patients. To archive time-dependent effects after pro-inflammatory stimulation in accordance with our patient study design, simulated samples we compared to untreated controls at one time point (0h). Thus we cannot fully exclude potentially small deviations due to time-dependent induction/suppression of biosynthesis of lipid mediators purely caused by incubation at 37°C. However, as strong induction/suppression of the relevant pro-inflammatory lipids after stimulation with LPS are well documented and because of the heterogeneity of the samples originating from different donors broadening the scatter of measured control values such effects should be negligible. Furthermore, a large number of prior publications already confirm the findings from the LPS-assay. However, to achieve optimal comparability to the results from ICU patients, we decided repeating this LPS assay using the same analytic LC/MS-MS methodology for both conditions identified in this study.

A systematically evaluation of selected lipids with markedly higher patient numbers during sepsis according to actual definitions, acute kidney injury and extracorporeal procedures seems to be the next big step, while monitoring patients’ pharmacotherapy.

Regarding the question of impact and novelty of our study in this field of research, we conclude that some of our findings confirm other published studies investigating lipid profiles during sepsis and inflammation, predominantly in animal models and partly in patients. Thus, subsequent deeper studies on these mediators in specific diseases such as e.g. pneumogenic sepsis or postoperative heart failure are encouraged. However, our study is the first to provide insights into the dynamics of plasma lipid level changes during hospitalization and clinical events. Despite all limitations and technical problems, we were able to identify several new lipid-based mediators with potential pathophysiological relevance and possible value as biomarkers.

## 5 Conclusions

Based on these findings, the following main conclusions can be drawn. First, we confirmed increased or decreased levels of certain lipids during inflammatory periods. Moreover, in several cases, these lipid levels remained consistently outside the normal range for extended periods, irrespective of any clinical event. Thus, our study strongly questions the proposed use of these lipids as biomarkers for indicating a transition between clinical stages. However, these lipids may have general yet undefined pathophysiological relevance for critically ill patients.

We confirm the down- and upregulation of other types of lipids such as 1-AG, 2-AG and 6-keto-PGF_1alpha_. These lipids showed high dynamics during the observation period, displayed remarkably elevated plasma levels and might therefore function as highly volatile candidates for markers indicating transitions of clinical stage, such as induction of systemic inflammation, aggravated acute kidney injury or (right) heart failure.

Taken in its entirety, our comprehensive pilot study provides extended and deeper insights into the role of lipid signaling in hospitalized ICU patients after cardiac surgery. Our study strongly encourages more targeted studies including a higher number of patients allowing sub-stratifications analyses and evaluation of the function and use of selected lipids as players and potential biomarkers in the critically ill.

## Data availability statement

The raw data supporting the conclusions of this article will be made available by the authors, without undue reservation.

## Ethics statement

The study design was approved by the local ethics committee at Frankfurt University Hospital (AZ: 265/09 and 476/13). The ethics committee at Frankfurt University Hospital also granted written approval of an extension of the studies mentioned herein including the determination of hospitalized patients’ lipids in plasma. The ethics committee also agreed in writing to the experiments using LPS-stimulated whole blood and the subsequent analysis of lipid mediators.

## Author contributions

HN, JR, AU, DS, GG, KZ, PP, ES, MP, PM, EU and TJM contributed to the conception or design of the work and sample acquisition; JR, UH, CA, NF, RG, ES, PM, TJM, VR and TB contributed to the acquisition, analysis, or interpretation of data; and JR, AU, UH, DS, MP, NF, PP, GG, ES, TB, VR and TJM have drafted the work or substantively revised it. All authors contributed to the article and approved the submitted version.
