# Supplementary material for: Analysis of fatty acid-derived lipids in critically ill patients after cardiac surgery yields novel pathophysiologically relevant mediators with possible relevance for systemic inflammatory reactions
Source: Front Immunol. 2025 Jan 7;15:1148806. doi: 10.3389/fimmu.2024.1148806 (PMC11826806; doi:10.3389/fimmu.2024.1148806)
Supplement: Supplementary file 1 [file DataSheet1.pdf]

Table 1 supplemental: LPS assays

Lipid	6 h vs control	16 h vs control	24 h vs control	Effect
Cer (d18:0/16:0)	p=0.001953**	p=0.001953**	p=0.001953**	↑
Cer (d18:0/18:0)	p=0.695312	p=0.001953**	p=0.001953**	↑
Cer (d18:0/24:0)	p=0.130859	p=0.375000	p=0.625000	●
Cer (d18:1/14:0)	p=0.769531	p=0.769531	p=0.130859	●
Cer (d18:1/16:0)	p=0.232422	p=0.001953**	p=0.001953**	↑
Cer (d18:1/18:0)	p=0.431641	p=0.005859**	p=0.001953**	↑
Cer (d18:1/18:1)	p=1.000000	p=0.528612	p=0.032969*	↑ (●)
Cer (d18:1/20:0)	p=0.105469	p=0.001953**	p=0.009766**	↑
Cer (d18:1/24:0)	p=1.000000	p=0.492188	p=0.048828*	↓ (●)
Cer (d18:1/24:1)	p=0.232422	p=0.845703	p=1.000000	●
Cer (d18:0/24:1)	p=0.921875	p=0.769531	p=0.322266	●
GlcCer (d18:1/16:0)	p=0.193359	p=1.000000	p=0.625000	●
GlcCer (d18:1/18:0)	p=0.322266	p=0.375000	p=0.492188	●
GlcCer (d18:1/24:1)	p=1.000000	p=0.431641	p=0.064453	●
LacCer (d18:1/16:0)	p=0.695312	p=0.048828*	p=0.625000	↑ (●)
LacCer (d18:1/18:0)	p=0.695312	p=0.001953**	p=0.130859	↑
LacCer (d18:1/24:0)	p=1.000000	p=0.027344*	p=0.048828*	↑ (●)
LacCer (d18:1/24:1)	p=0.625000	p=0.013672*	p=0.492188	↑
SPH d18:0	p=0.556641	p=0.005889**	p=0.001953**	↑
S1P d18:0	p=0.275391	p=0.001953**	p=0.001953**	↓
SPH d18:1	p=0.048828*	p=0.001953**	p=0.001953**	↑
S1P d18:1	p=0.358639	p=0.375000	p=0.001953**	↓
LTB4_195	---	---	---	---
PGE2	p=0.048828*	p=0.001953**	p=0.009766**	↑
PGD2	p=0.064453	p=0.001953**	p=0.001953**	↑
PGF2alpha	p=0.013672*	p=0.019531*	p=0.037109*	↑
6-keto-prostaglandin F1alpha	p=1.000000	p=1.000000	p=1.000000	●
Thromboxane B2	p=0.064453	p=0.921875	p=0.083984	●
5-HETE_115	---	---	---	---
12-HETE_179	---	---	---	---
15-HETE_219	---	---	---	---
20-HETE_289	---	---	---	---
56 DHET_145	---	---	---	---
89 DHET_127	---	---	---	---
1112 DHET_167	---	---	---	---
1415 DHET_207	---	---	---	---
AEA	p=0.275391	p=0.001953**	p=0.003906**	↑
OEA	p=0.375000	p=0.001953**	p=0.001953**	↑
PEA	p=0.232422	p=0.001953**	p=0.001953**	↑
1-AG	p=1.000000	p=0.322266	p=0.492188	●
2-AG	p=0.845703	p=0.431641	p=0.160156	●
LPA (16:0)	p=0.195313	p=0.003906**	p=0.003906**	↑
LPA (18:0)	p=0.546875	p=0.007812**	p=0.003906**	↑

LPA (18:1)	p=0.460938	p=0.003906**	p=0.003906**	↑
LPA (18:2)	p=0.195313	p=0.007812**	p=0.019531*	↑
LPA (18:3)	p=0.843750	p=0.003906**	p=0.003906**	↑
LPA (20:4)	p=0.382813	p=0.003906**	p=0.003906**	↑
910 EpOME_171	---	---	---	---
1213 EpOME_113	---	---	---	---
910 DiHOME_201	---	---	---	---
1213 DiHOME_129	---	---	---	---
9-HODE_141	---	---	---	---
13-HODE_113	---	---	---	---

↑ = Lipid significantly upregulated, ↓= Lipid significantly downregulated, ● = Lipid not regulated, --- = no data available. * $p < 0.05$; ** $p < 0.01$. LPS data were available for 38 of the 53 investigated lipids. For the LPS assays, a lipid was defined as significantly regulated, if p <0.05 for any of the three time points (6, 16 and 24 h) vs control. No significance correction was applied because this was seen as a time series and it was not important here at which (or on how many) of the three time points a lipid was significantly regulated vs control. Three (pairwise with respect to the 10 probands) two-dependent-sample Wilcoxon-Mann-Whitney tests for each control vs 6, 16 and 24 h were applied without significance correction. Even in the case of significance correction (Bonferroni, p < 0.0167), only four (Cer (d18:1/18:1), Cer (d18:1/24:0), LacCer (d18:1/16:0), LacCer (d18:1/24:0)) of the formerly 27 regulated lipids would remain unregulated (marked in the table additionally as (●)).
