# Supplementary material for: Analysis of fatty acid-derived lipids in critically ill patients after cardiac surgery yields novel pathophysiologically relevant mediators with possible relevance for systemic inflammatory reactions
Source: Front Immunol. 2025 Jan 7;15:1148806. doi: 10.3389/fimmu.2024.1148806 (PMC11826806; doi:10.3389/fimmu.2024.1148806)
Supplement: Supplementary file 2 [file Presentation1.pptx]

## Slide 1
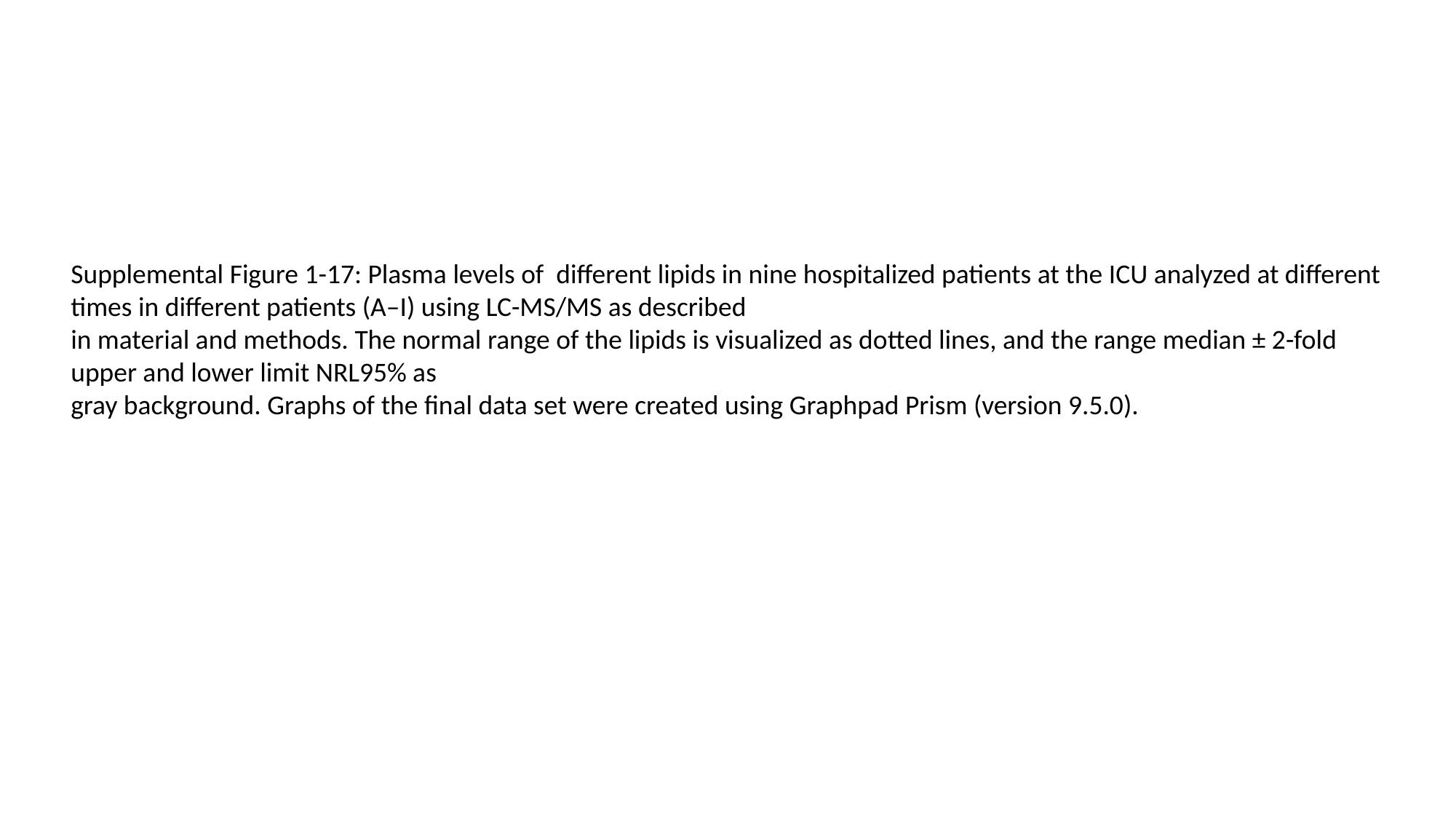

Supplemental Figure 1-17: Plasma levels of different lipids in nine hospitalized patients at the ICU analyzed at different times in different patients (A–I) using LC-MS/MS as described
in material and methods. The normal range of the lipids is visualized as dotted lines, and the range median ± 2-fold upper and lower limit NRL95% as
gray background. Graphs of the final data set were created using Graphpad Prism (version 9.5.0).

## Slide 2
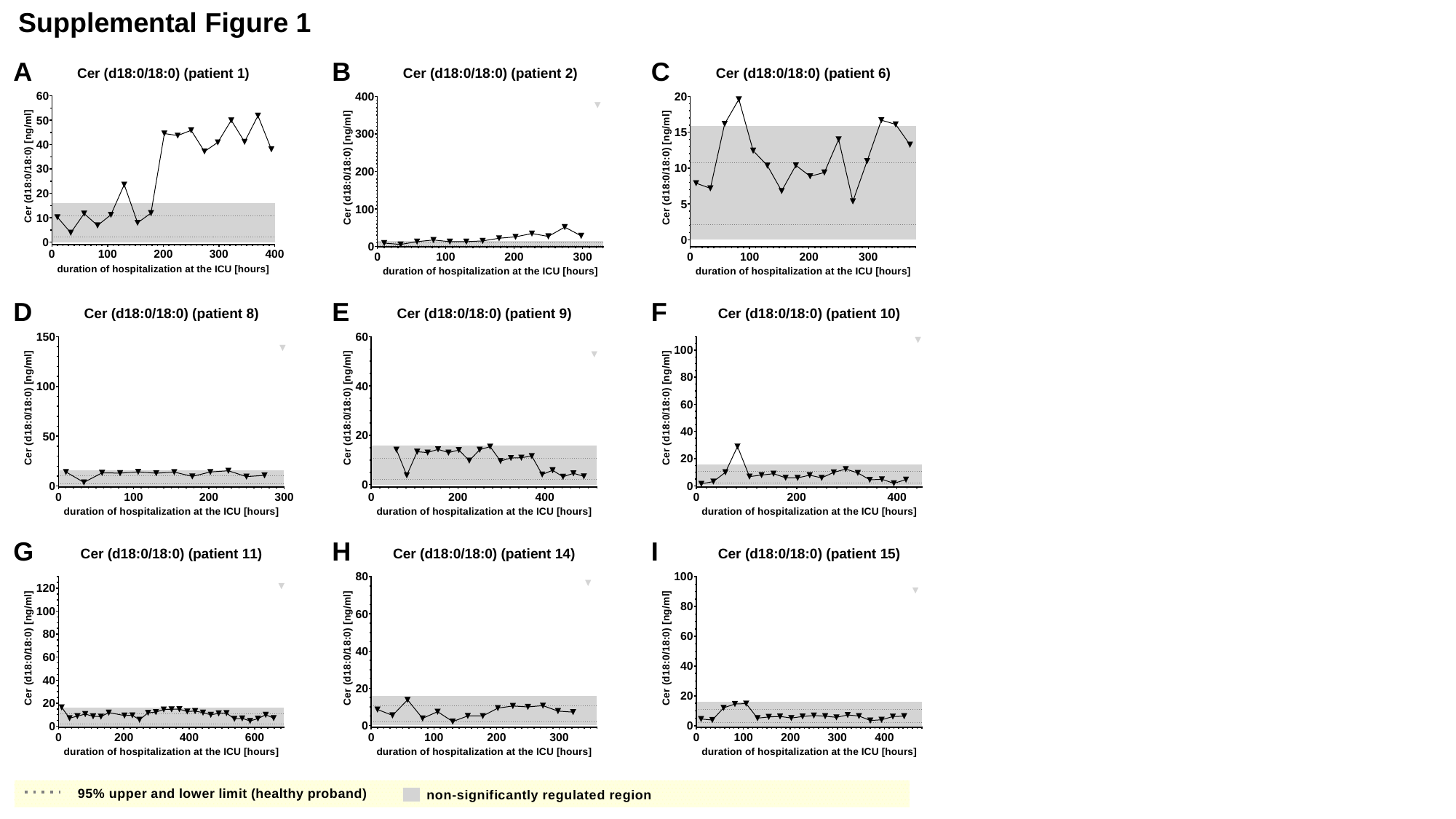

Supplemental Figure 1

## Slide 3
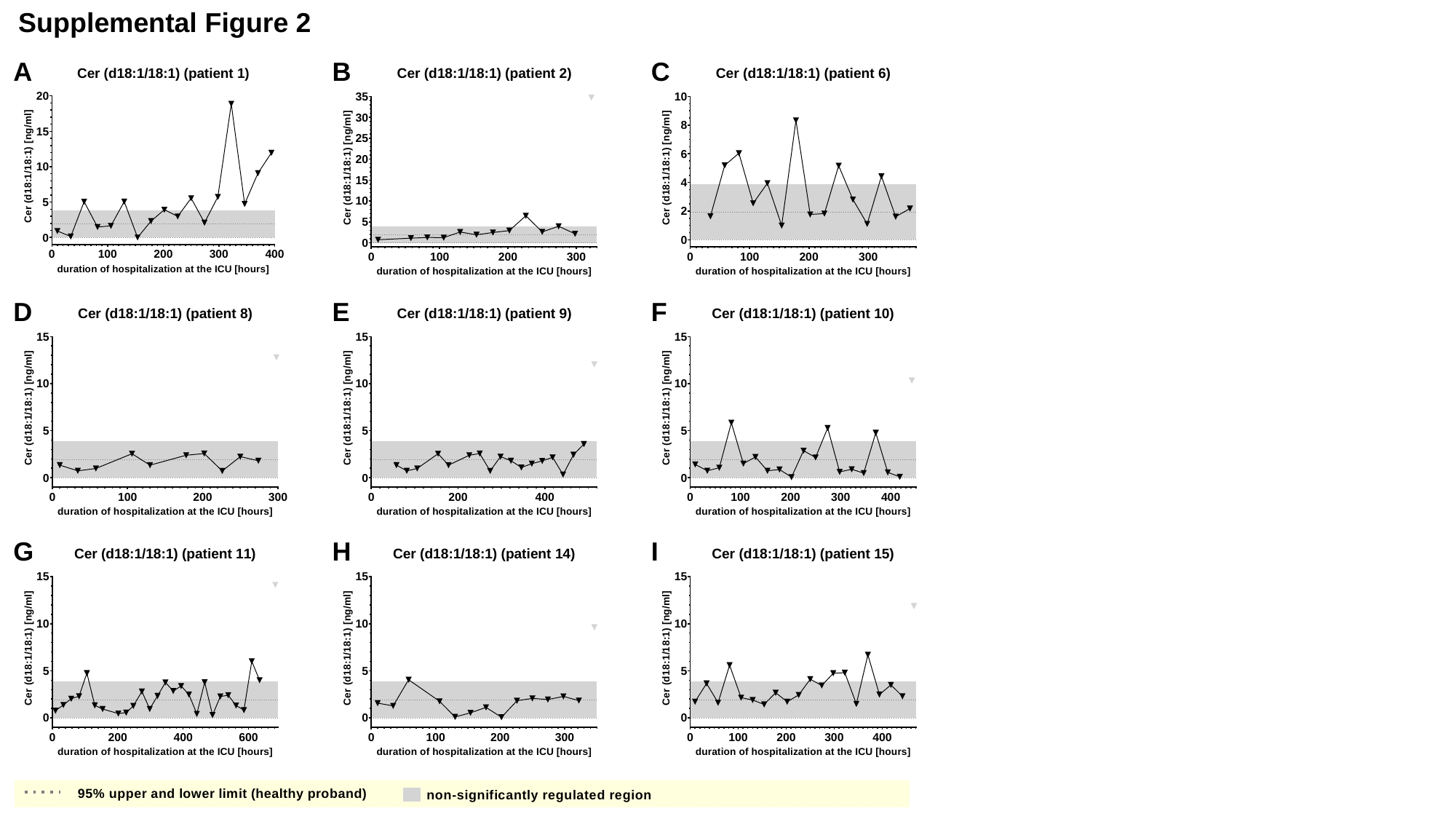

Supplemental Figure 2

## Slide 4
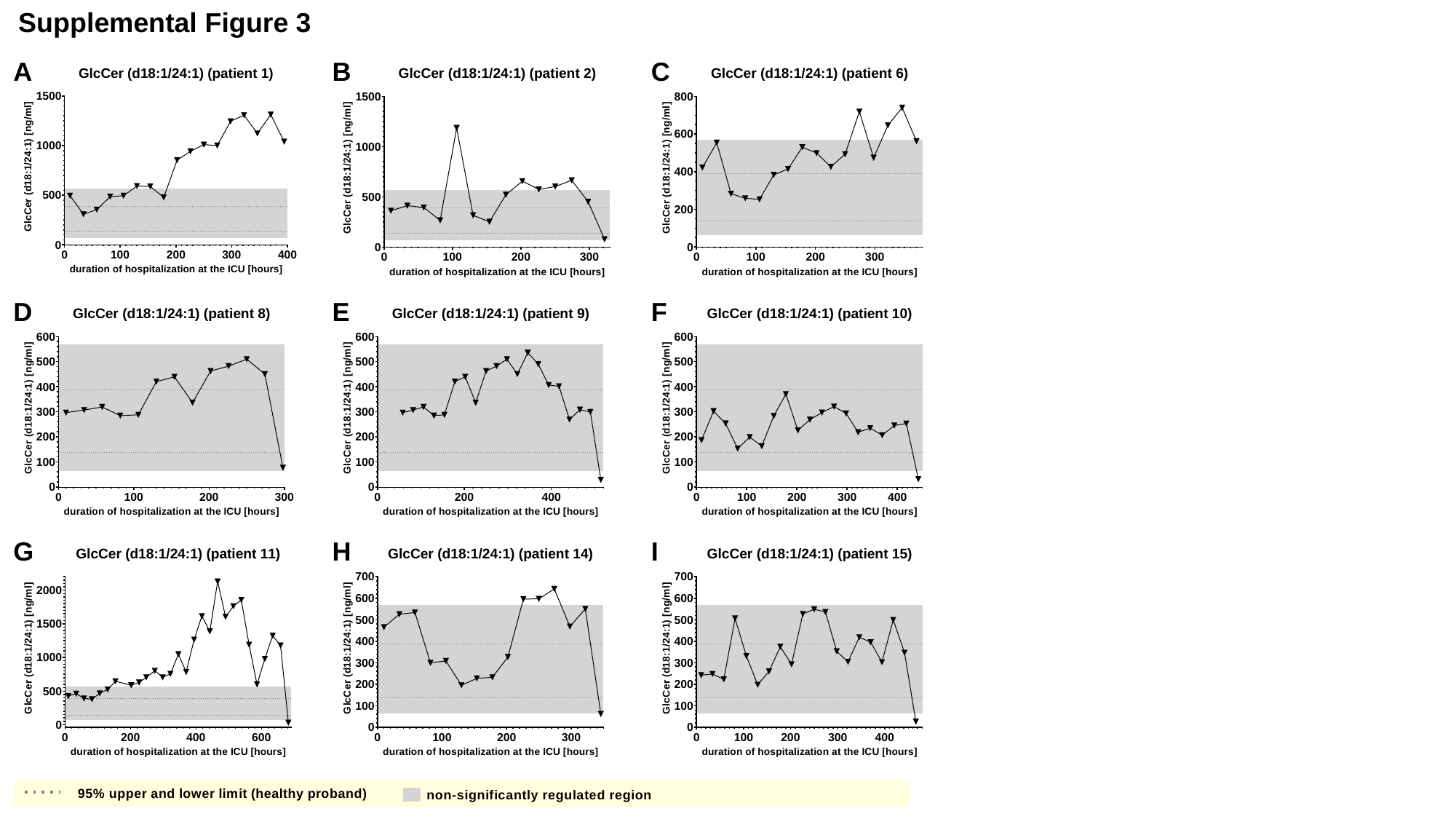

Supplemental Figure 3

## Slide 5
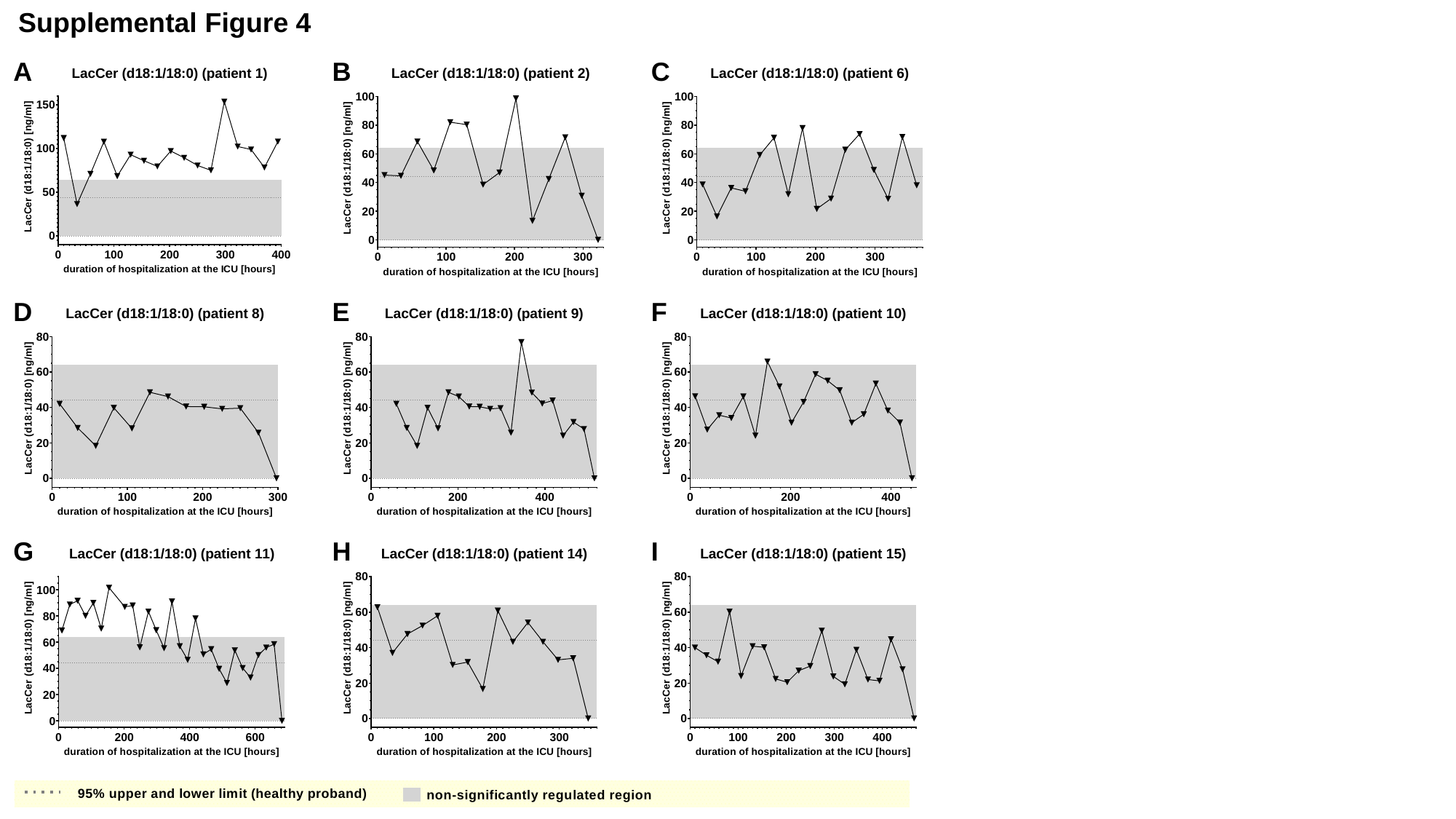

Supplemental Figure 4

## Slide 6
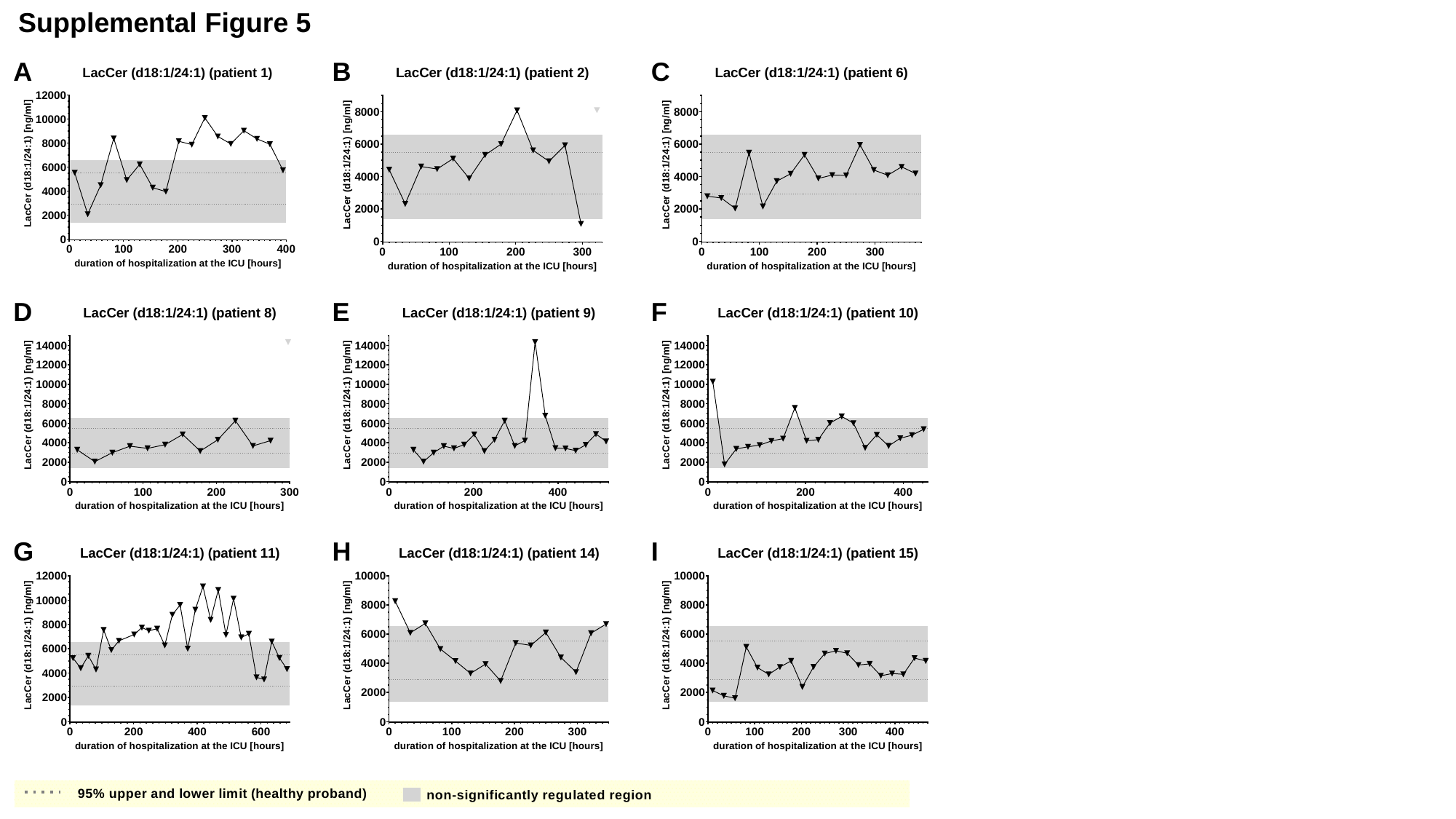

Supplemental Figure 5

## Slide 7
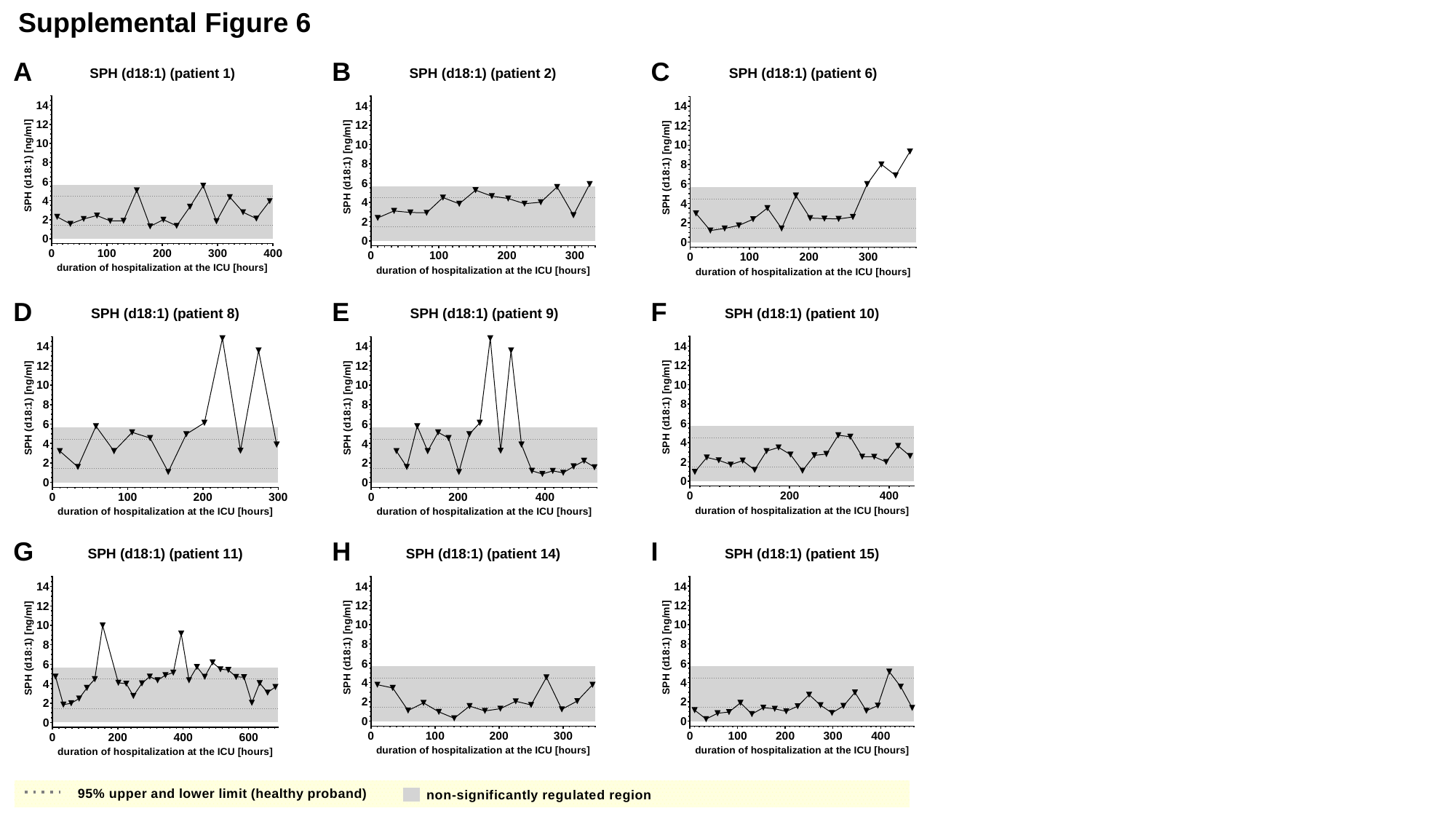

Supplemental Figure 6

## Slide 8
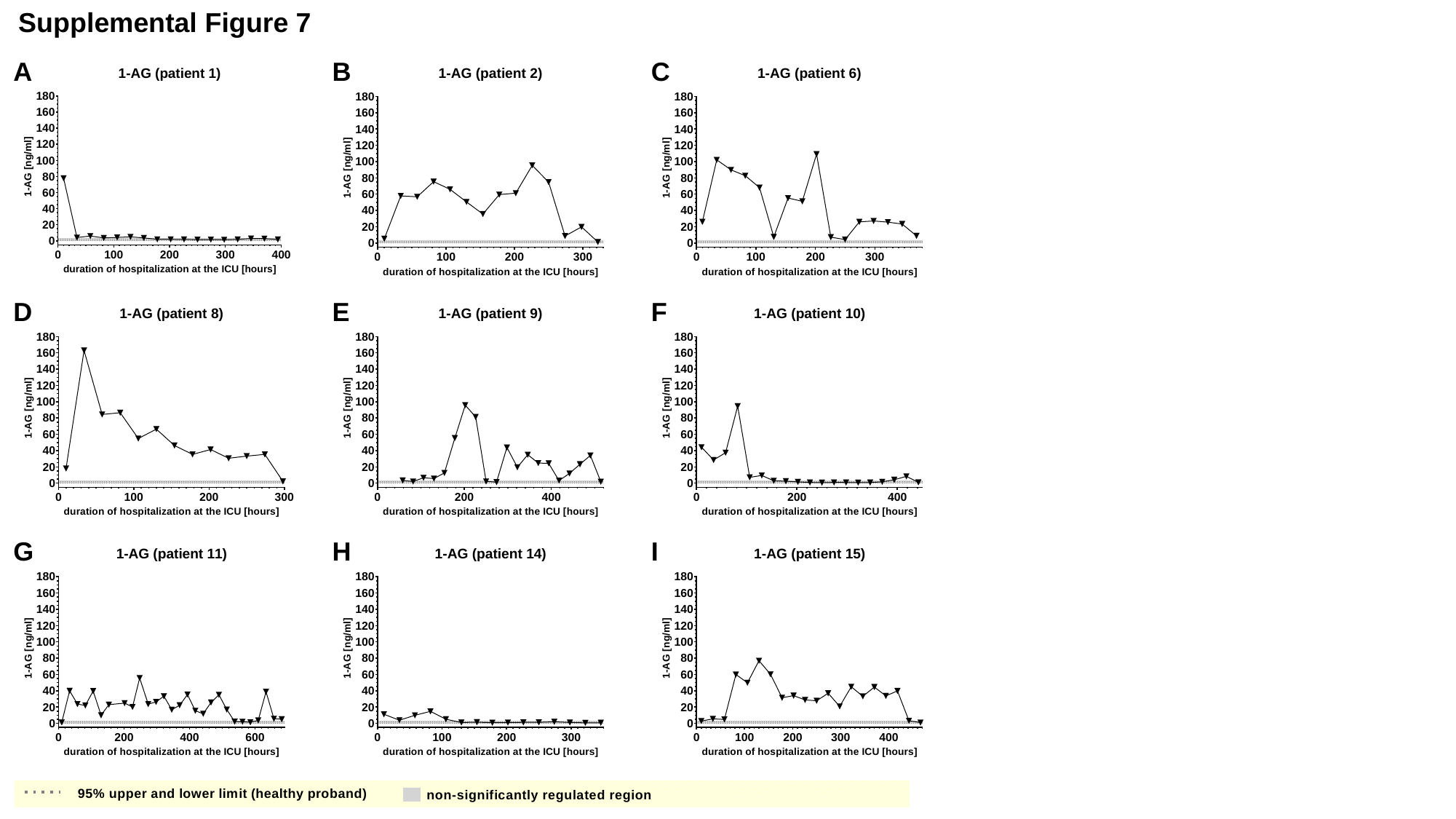

Supplemental Figure 7

## Slide 9
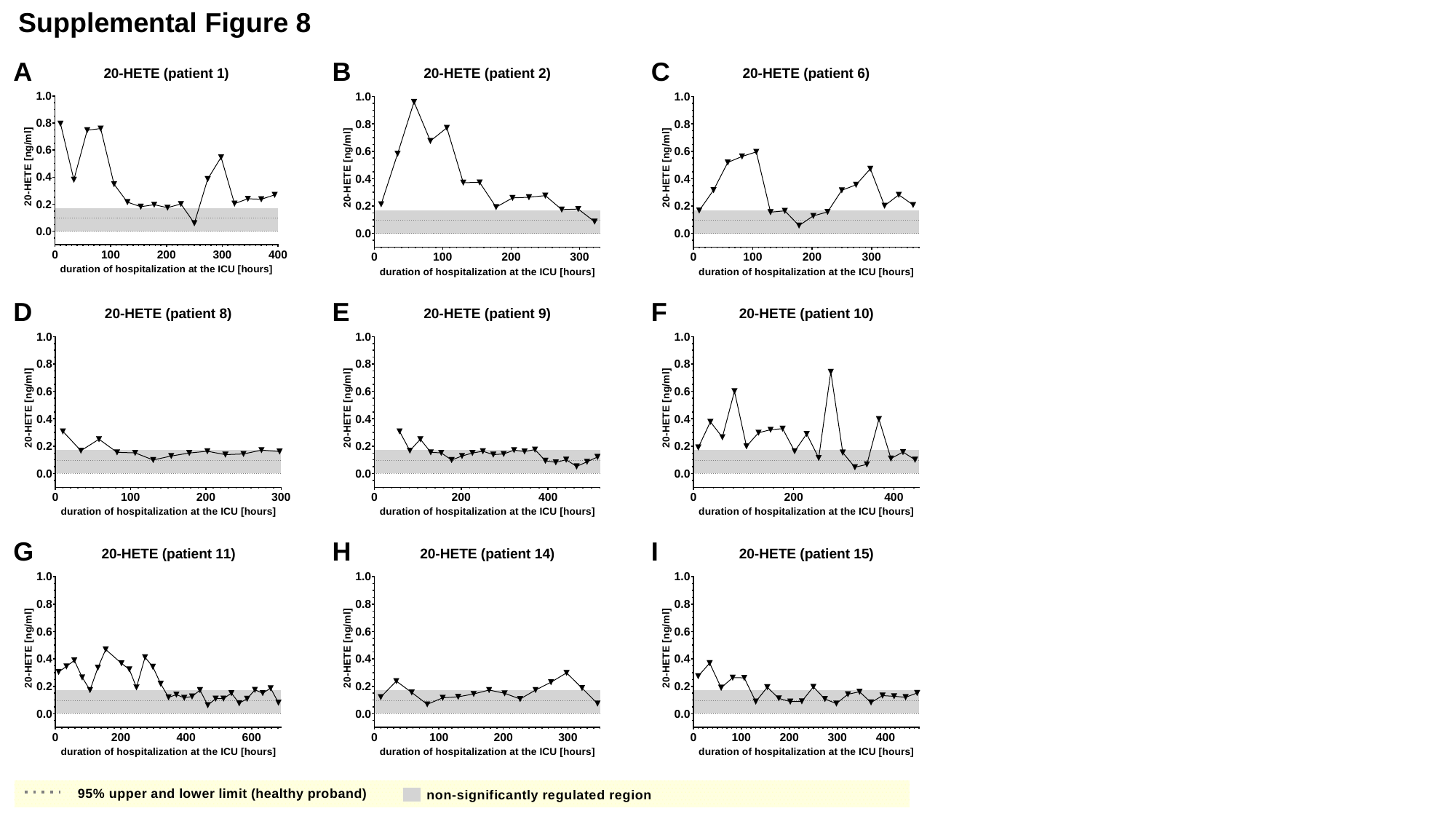

Supplemental Figure 8

## Slide 10
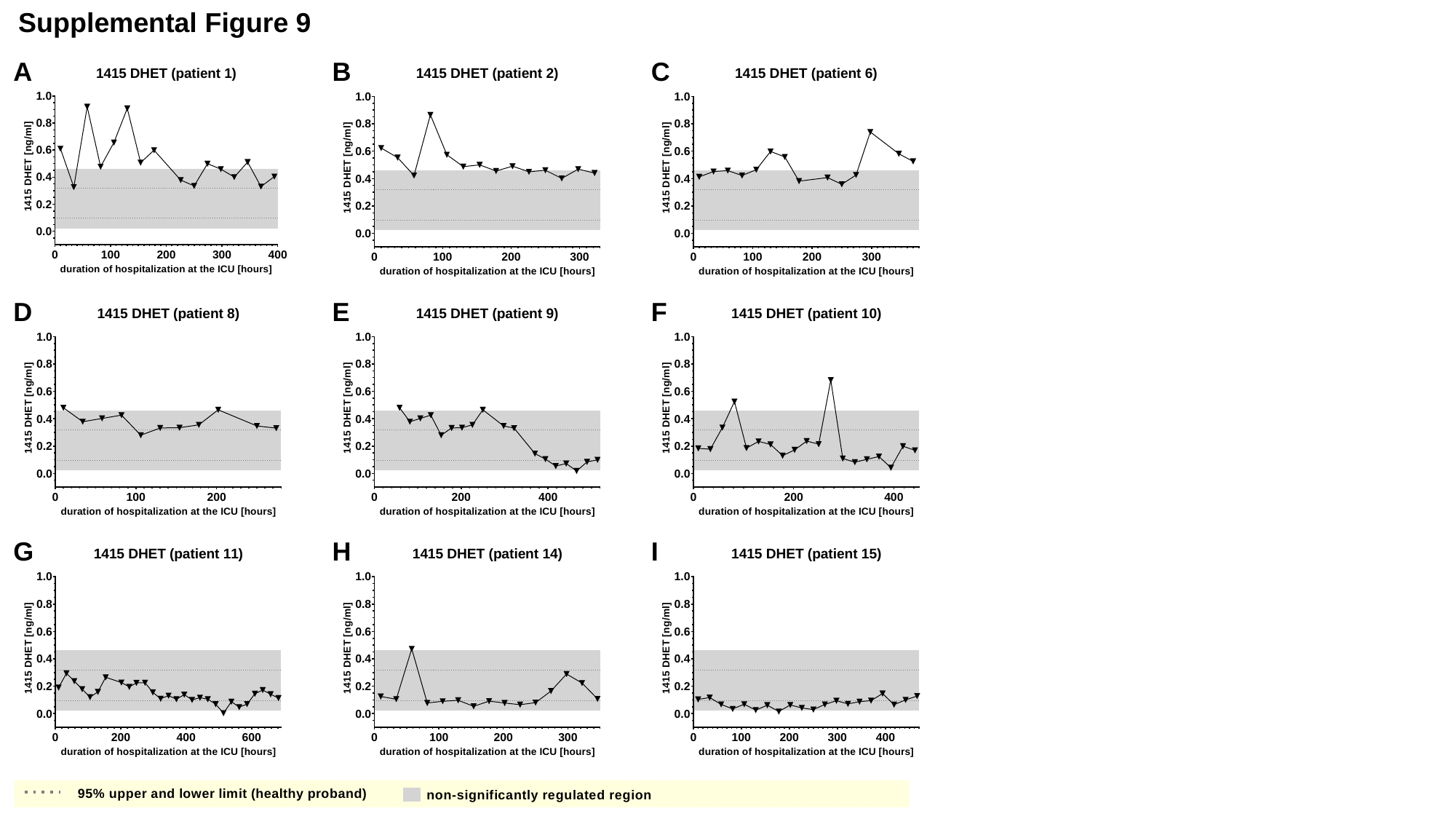

Supplemental Figure 9

## Slide 11
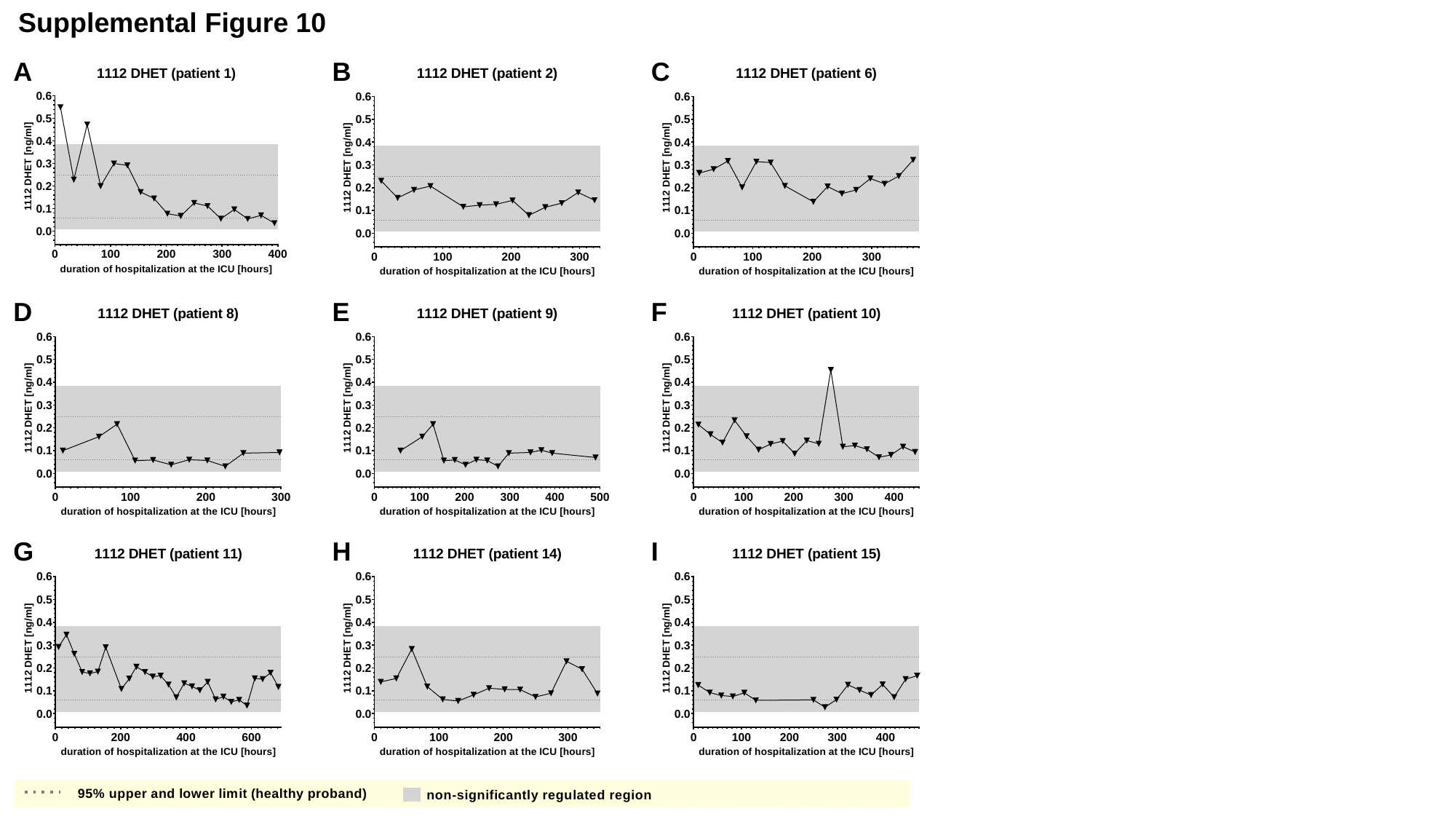

Supplemental Figure 10

## Slide 12
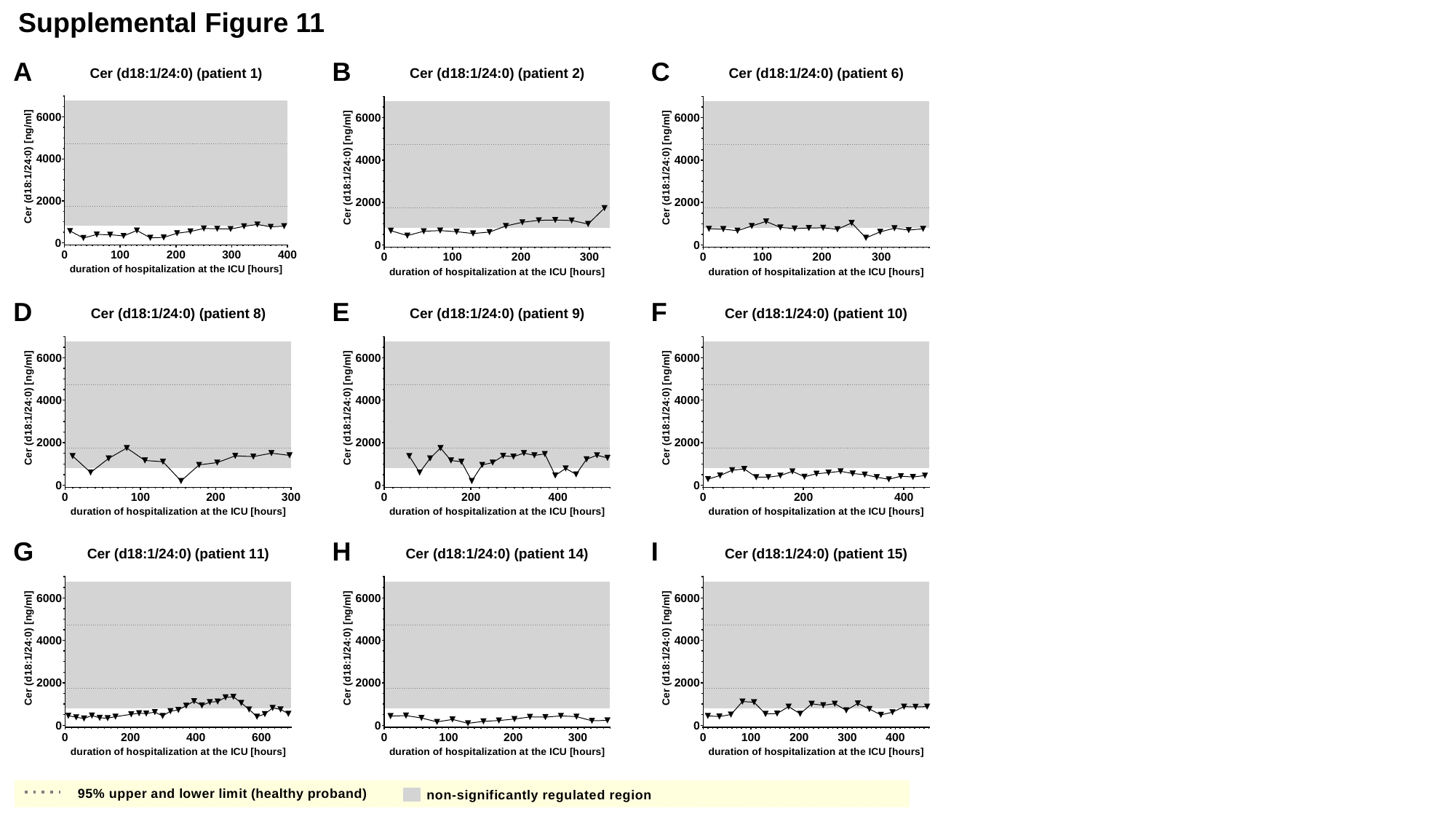

Supplemental Figure 11

## Slide 13
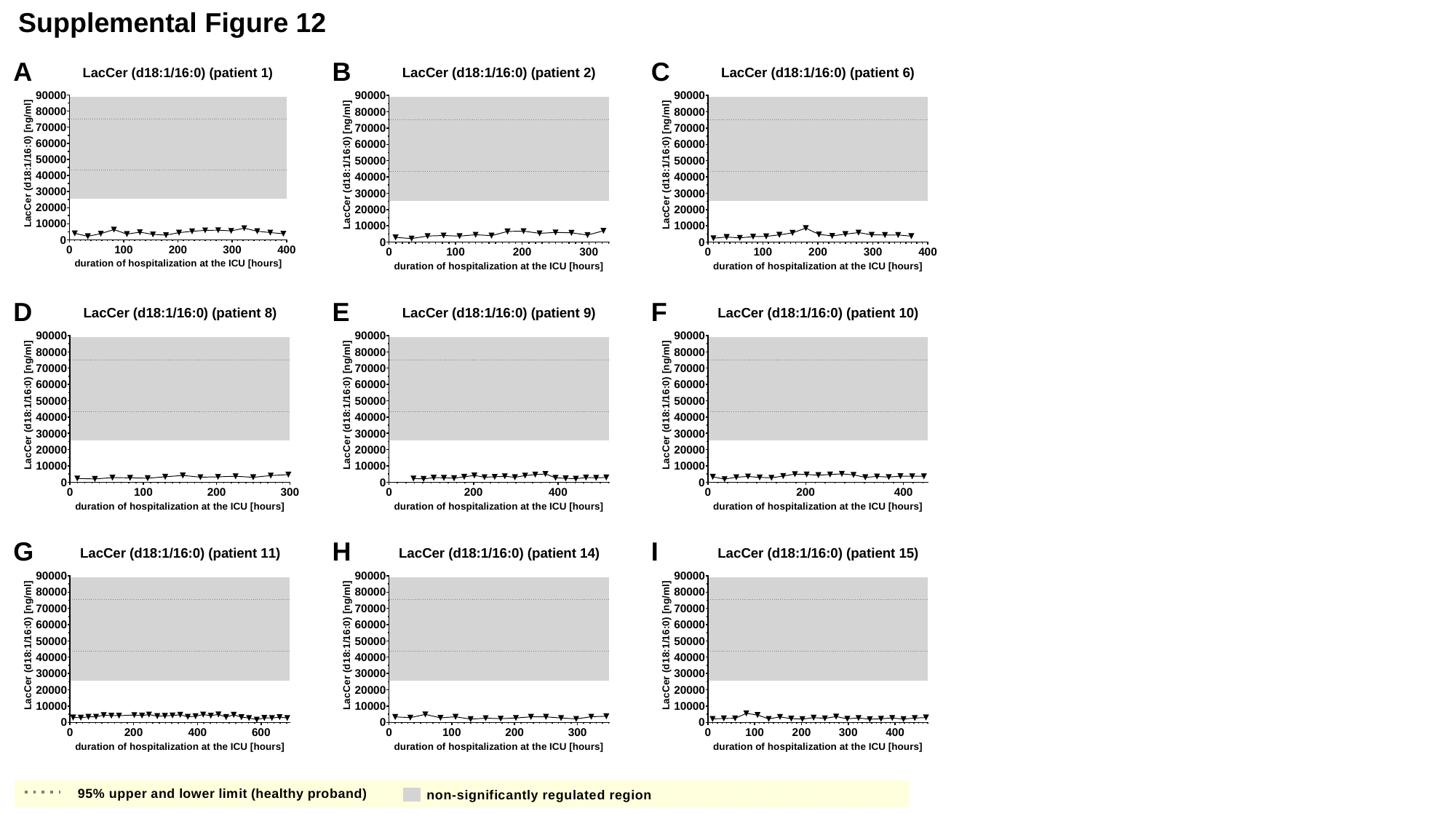

Supplemental Figure 12

## Slide 14
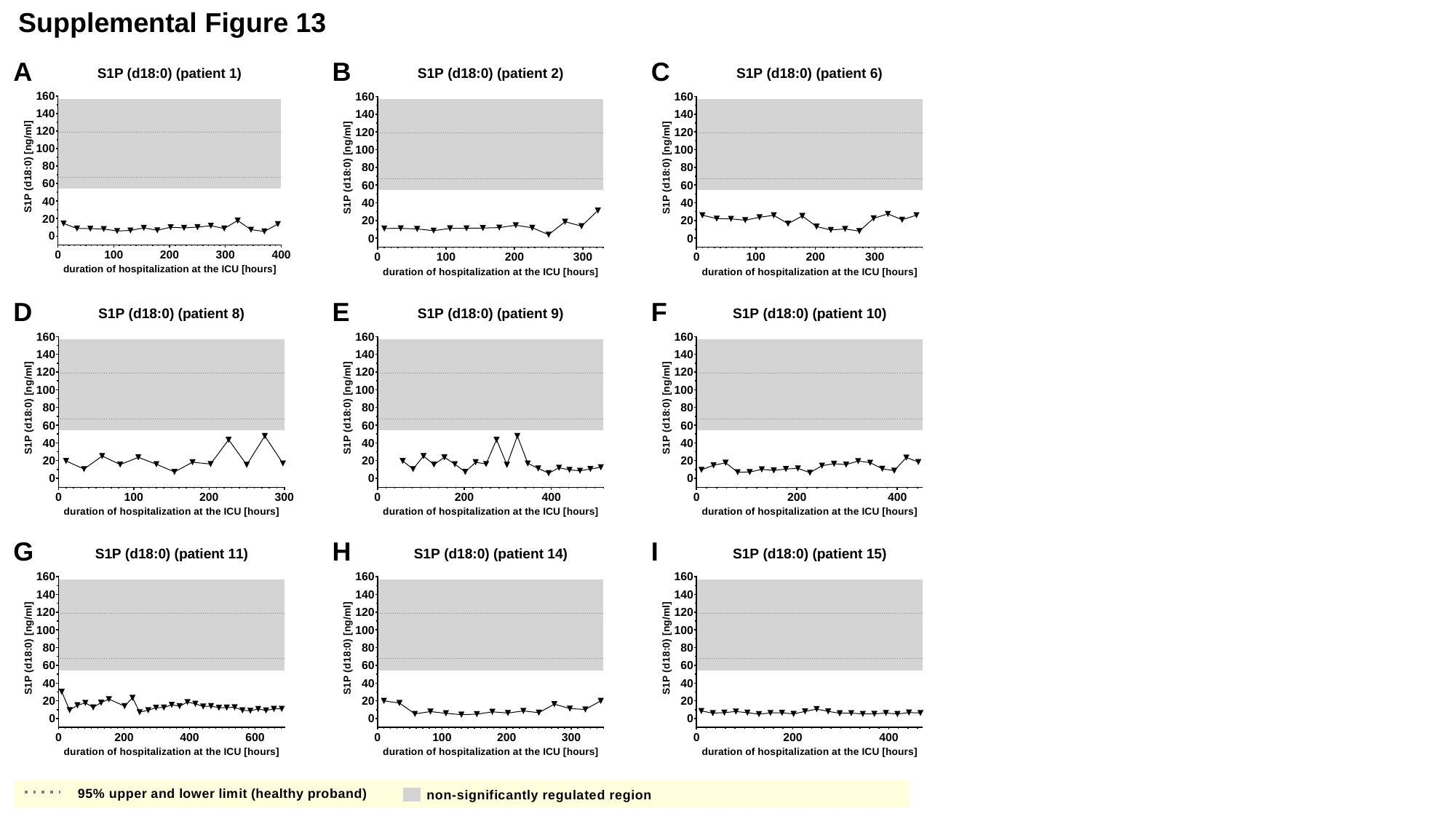

Supplemental Figure 13

## Slide 15
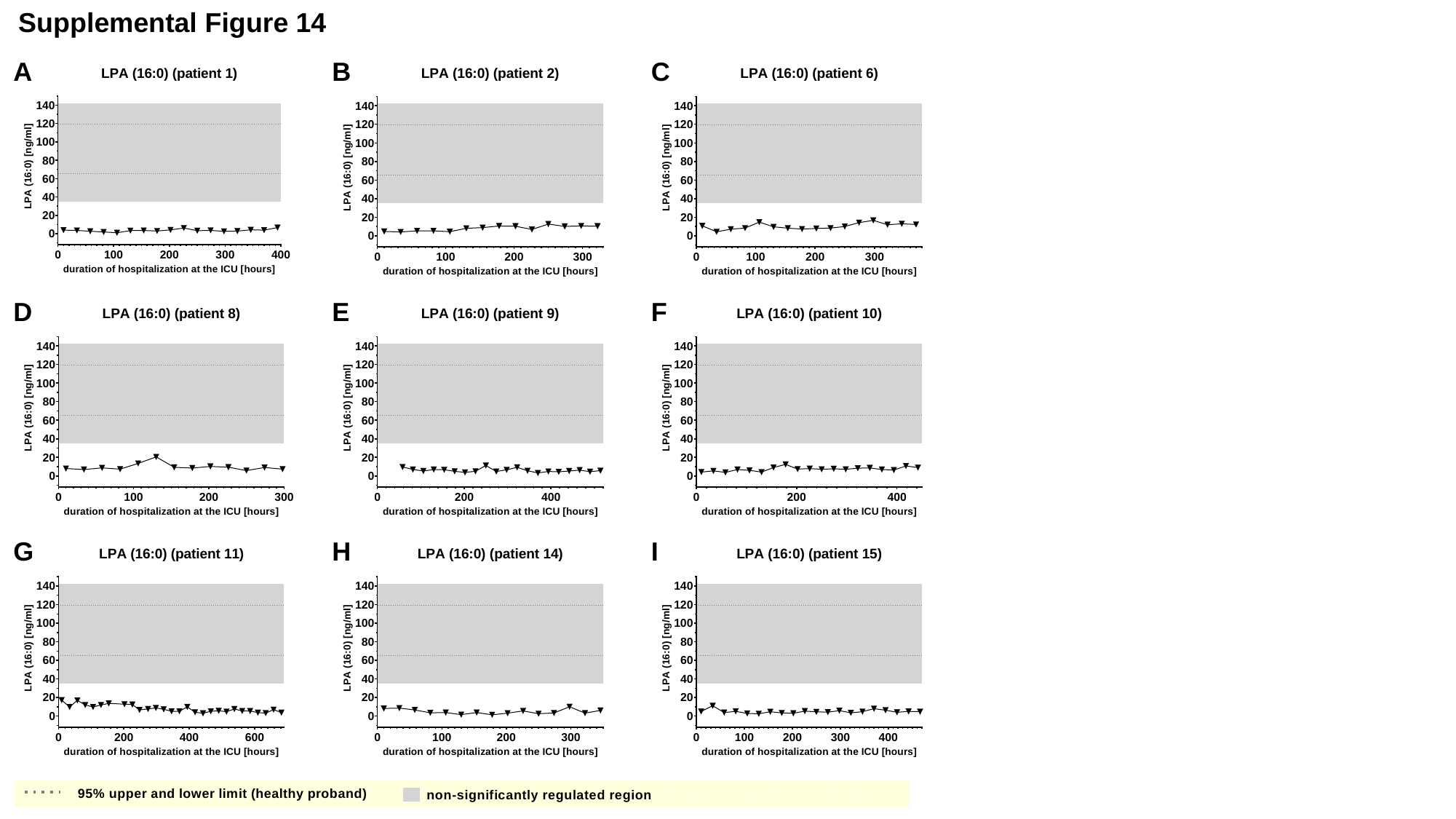

Supplemental Figure 14

## Slide 16
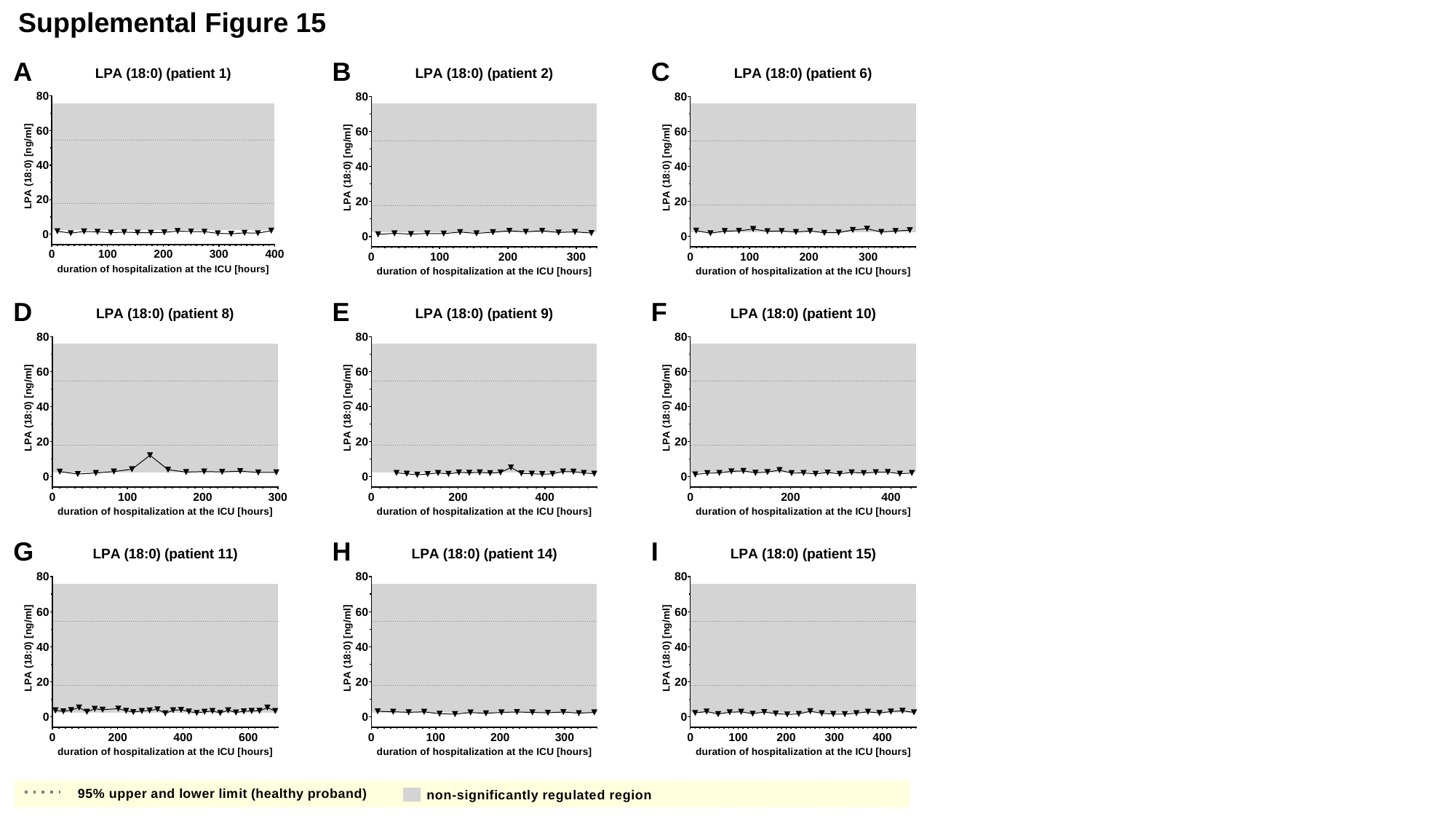

Supplemental Figure 15

## Slide 17
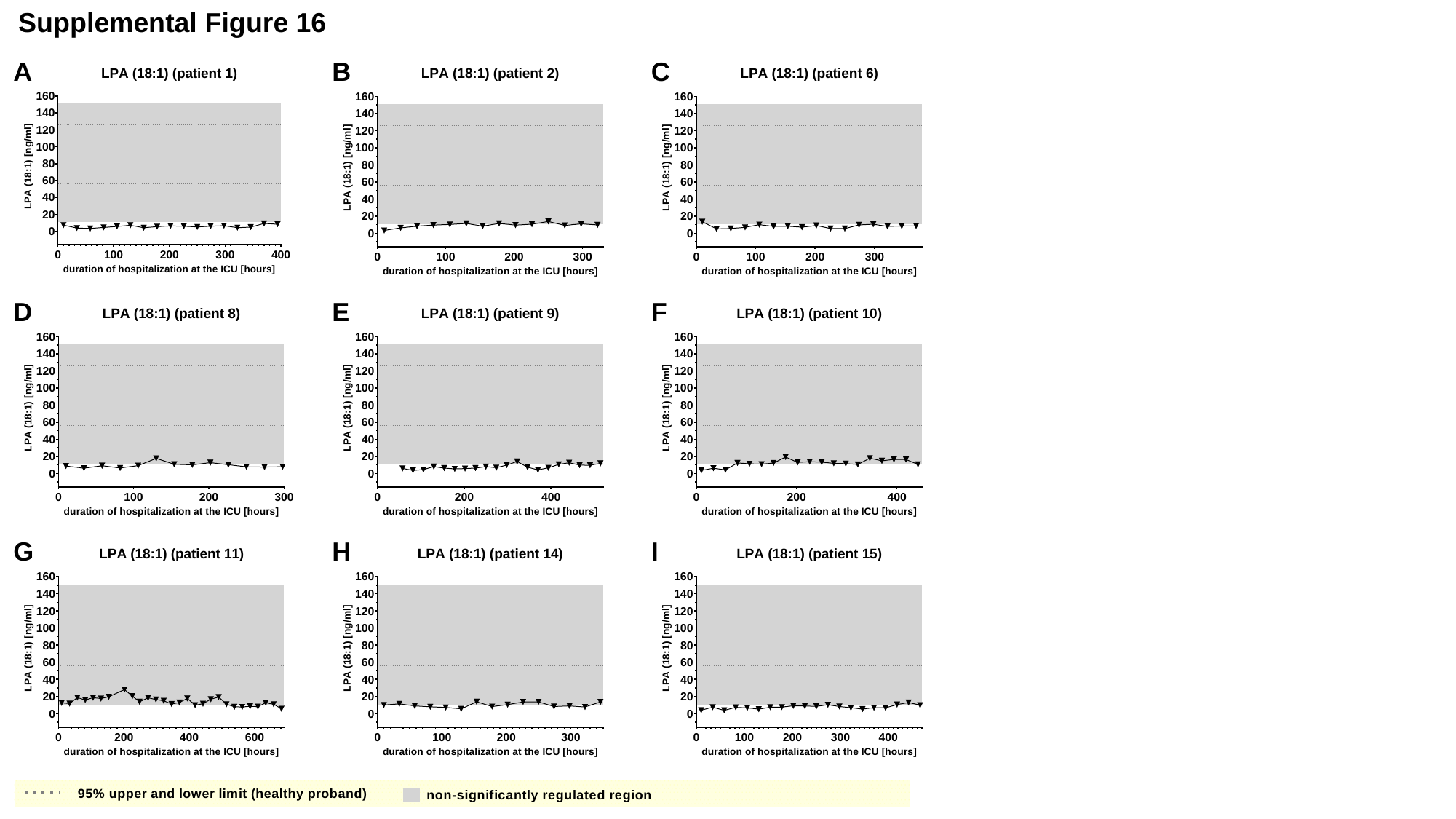

Supplemental Figure 16

## Slide 18
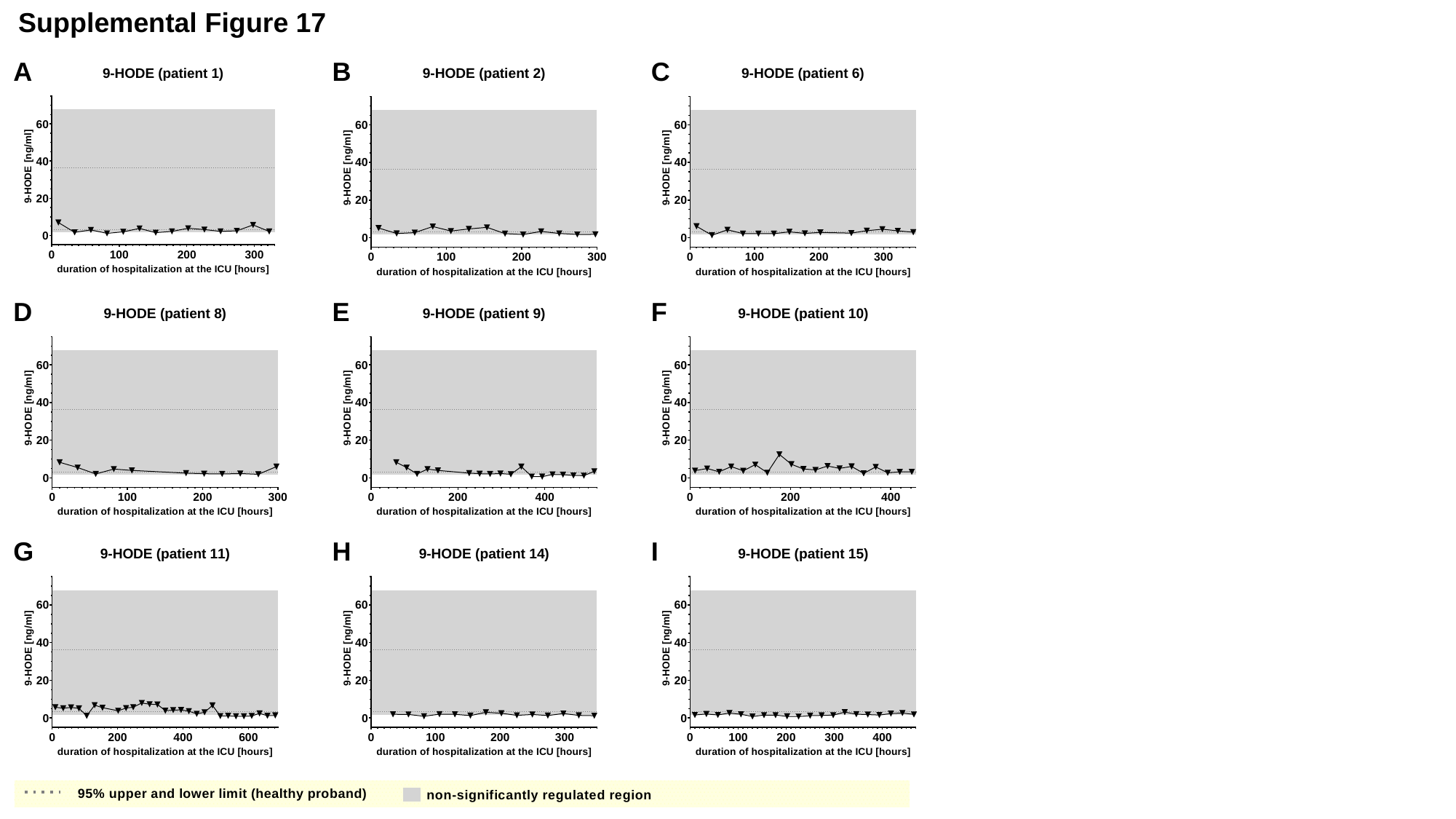

Supplemental Figure 17

## Slide 19
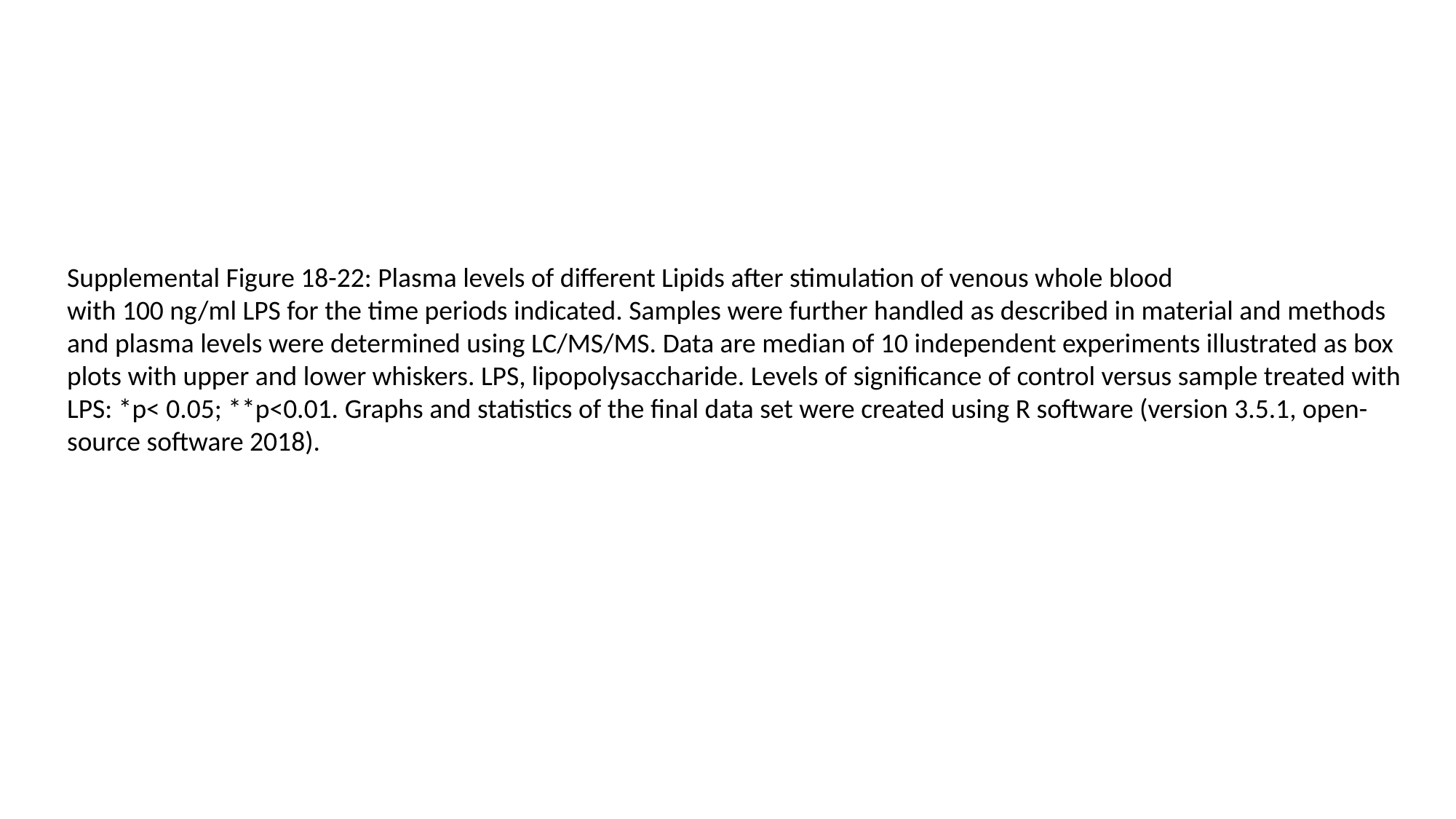

Supplemental Figure 18-22: Plasma levels of different Lipids after stimulation of venous whole blood
with 100 ng/ml LPS for the time periods indicated. Samples were further handled as described in material and methods and plasma levels were determined using LC/MS/MS. Data are median of 10 independent experiments illustrated as box plots with upper and lower whiskers. LPS, lipopolysaccharide. Levels of significance of control versus sample treated with LPS: *p< 0.05; **p<0.01. Graphs and statistics of the final data set were created using R software (version 3.5.1, open-source software 2018).

## Slide 20
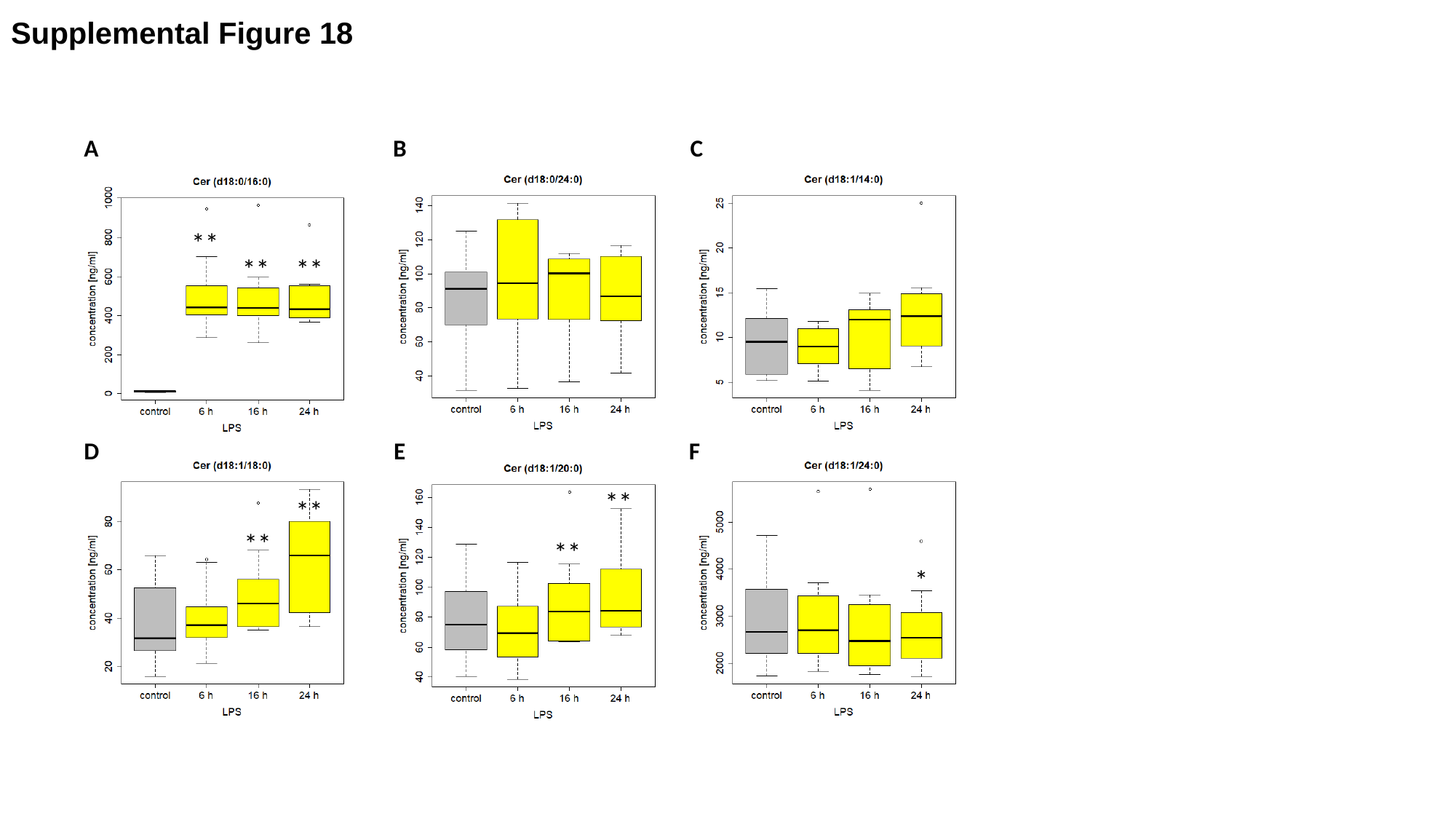

# Supplemental Figure 18
 A B C
**
**
**
 D E F
**
**
**
**
*

## Slide 21
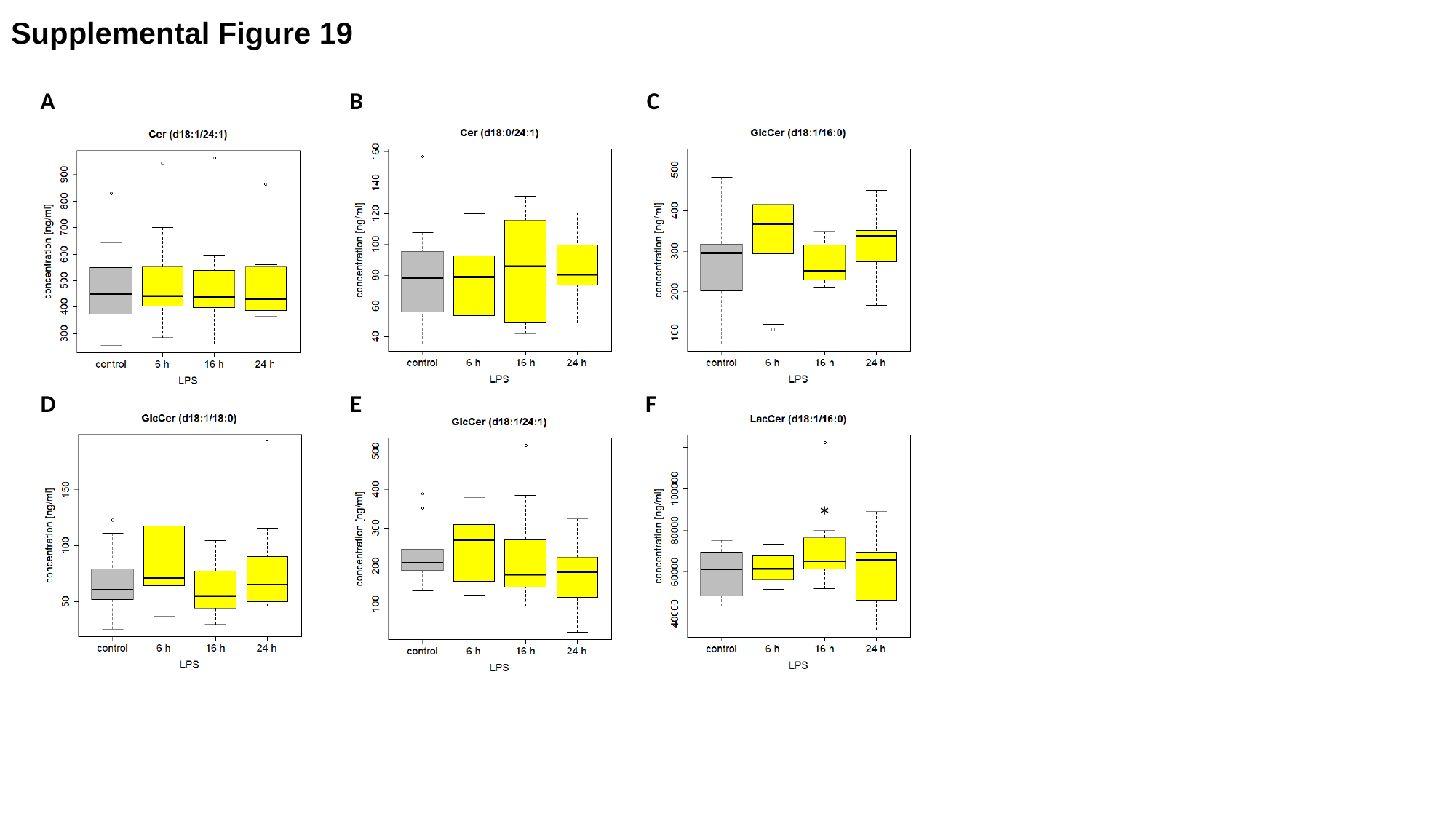

# Supplemental Figure 19
 A B C
 D E F
*

## Slide 22
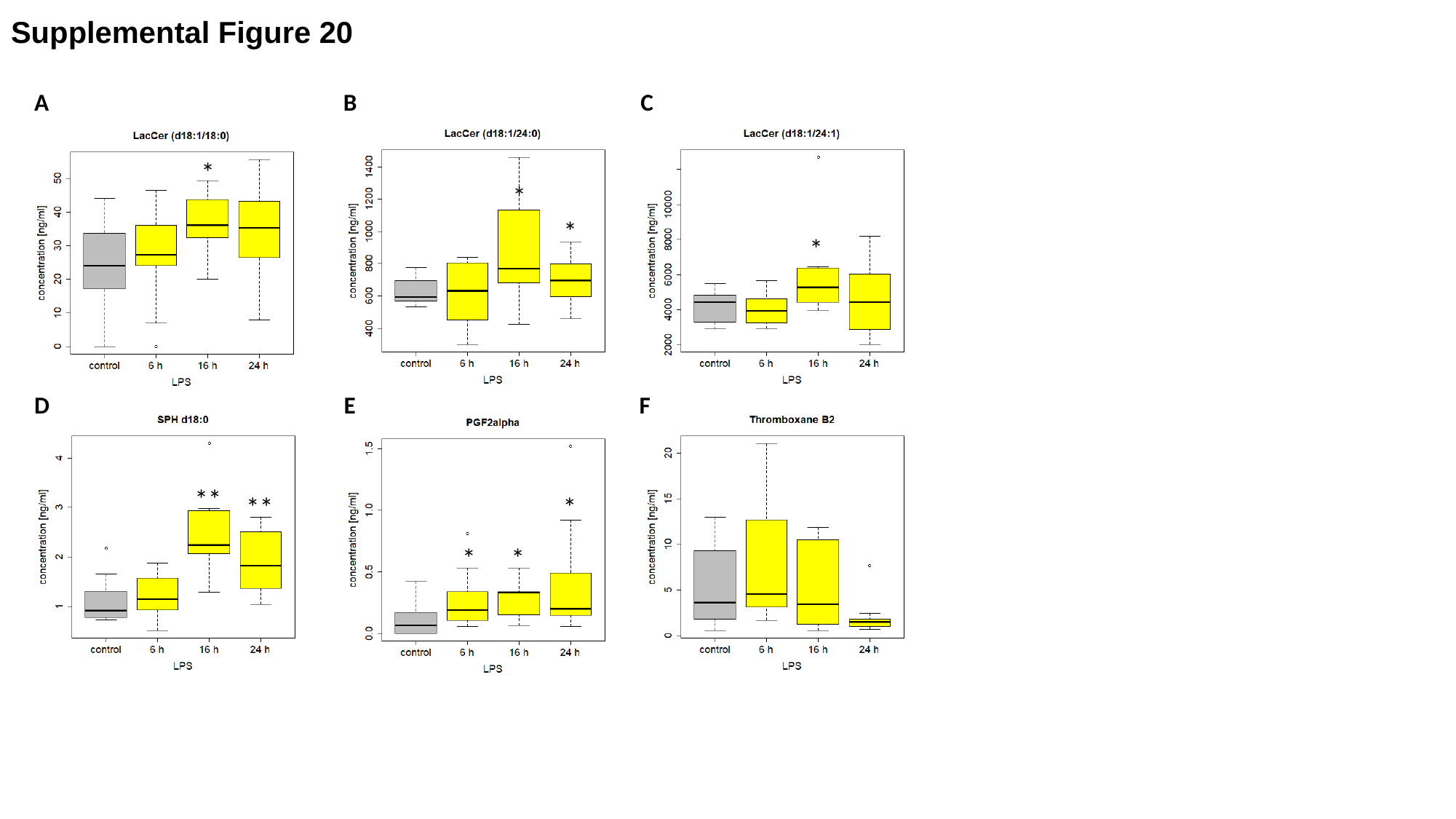

# Supplemental Figure 20
 A B C
*
*
*
*
 D E F
**
**
*
*
*

## Slide 23
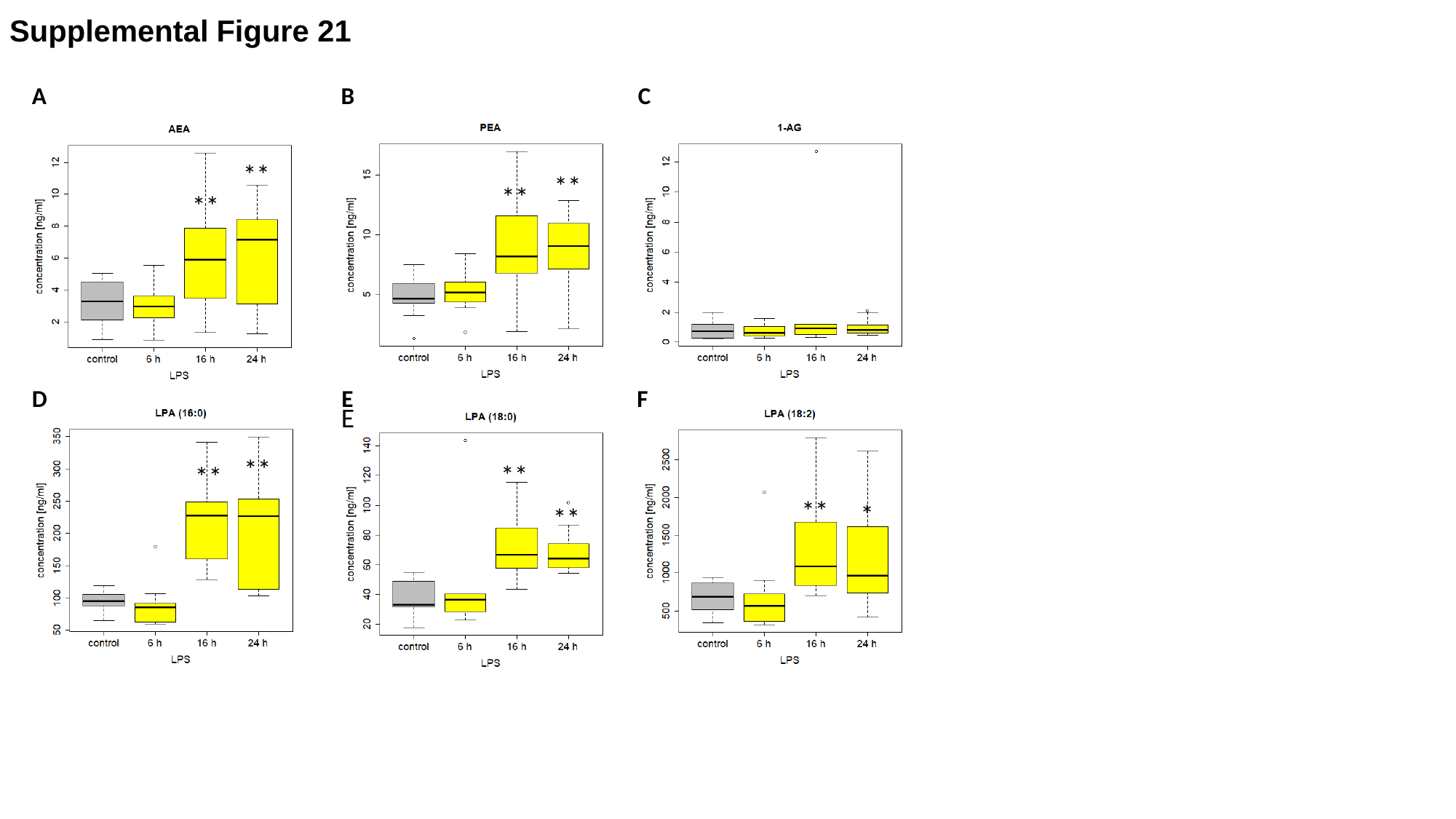

# Supplemental Figure 21
 A B C
**
**
**
**
 D E F
E
**
**
**
**
*
**

## Slide 24
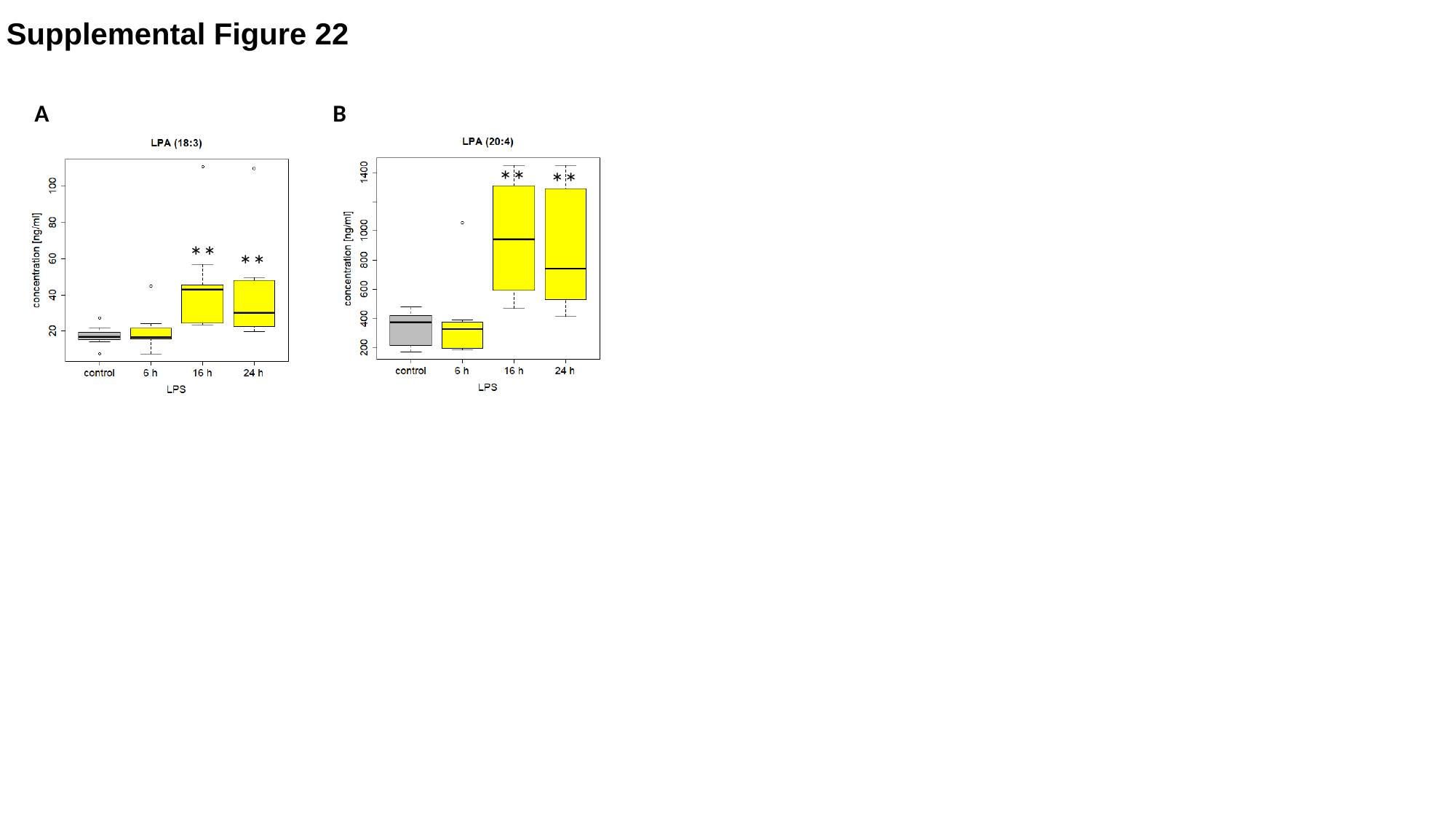

# Supplemental Figure 22
A B
**
**
**
**
